# Epithelial but not stromal expression of collagen alpha-1(III) is a diagnostic and prognostic indicator of colorectal carcinoma

**DOI:** 10.18632/oncotarget.6815

**Published:** 2016-01-03

**Authors:** Xiao-Qing Wang, Zu-Xiong Tang, Dong Yu, Shu-Jian Cui, Ying-Hua Jiang, Qian Zhang, Jie Wang, Peng-Yuan Yang, Feng Liu

**Affiliations:** ^1^ Department of Systems Biology for Medicine of School of Basic Medical Sciences, and Institutes of Biomedical Sciences, Fudan University, Shanghai, China; ^2^ Department of Chemistry, Fudan University, Shanghai, China; ^3^ Minhang Hospital, Fudan University, Shanghai, China; ^4^ College of Bioscience and Biotechnology, Key Laboratory of Crop Genetics and Physiology of Jiangsu Province, Yangzhou University, Yangzhou, Jiangsu, China; ^5^ Department of General Surgery, The First Affiliated Hospital of Soochow University, Suzhou, China; ^6^ Department of Plastic and Reconstructive Surgery, Shanghai Ninth People's Hospital, Shanghai Jiaotong University School of Medicine, Shanghai, China

**Keywords:** colorectal cancer, COL3A1, prognosis, protein marker

## Abstract

Colorectal cancer (CRC) is the third most common cancer in males and the second in females worldwide with very poor prognosis. *Collagen alpha-1(III)* (*COL3A1*) gene, encoding an extracellular matrix protein, is upregulated in human cancers. Here, we revealed that *COL3A1* was increased in CRC by analysis of five Oncomine gene expression datasets (*n* = 496). Immunohistochemistry analysis of a tissue microarray (*n* = 90) demonstrated that cancer epithelial but not stromal COL3A1 was significantly upregulated comparing with the normal counterparts. High *COL3A1* mRNA and/or protein expression was accompanied with high stage, T stage, Dukes stage, grade and older age, as well as smoking and recurrence status. Upregulated *COL3A1* predicted poor overall (*p* = 0.003) and disease-free (*p* = 0.025) survival. Increased epithelial but not stromal COL3A1 protein predicted worse outcome (*p* = 0.03). Older patients (age>65) with high *COL3A1* had worse survival than younger (age≤65) with high *COL3A1*. Plasma COL3A1 was increased in CRC patients (*n* = 86) by 5.4 fold comparing with healthy individuals, enteritis and polyps patients. Plasma COL3A1 had an area under curve (AUC) of 0.92 and the best sensitivity/specificity of 98.8%/69.1%. While plasma CEA had a poorer prediction power (AUC = 0.791, sensitivity/selectivity = 70.2%/73.0%). Older patients (age≥60) had higher plasma COL3A1 than younger patients. The epithelial COL3A1 protein had an AUC of 0.975 and the best sensitivity/specificity of 95.2%/91.1%. Silencing of *COL3A1* suppressed CRC cell proliferation in *in vitro* MTT assay and in *in vivo* Zebra fish xenograft model by downregulation of PI3K/AKT and WNT signaling. COL3A1 was a novel diagnosis and prognosis marker of CRC.

## INTRODUCTION

Colorectal cancer (CRC) is the third most common cancer in males and the second in females, with 746 and 614 thousand incidences in 2012 for both sexes, respectively, according to the GLOBOCAN statistics [1]. The overall mortality of this deadly cancer is 51% (694 thousand), indicating a very poor prognosis. In China, the total occurrence of CRC is 253 thousand in 2012, with a more serious mortality rate of 55% [1]. There is an urgent need to identify reliable markers for early diagnosis of CRC and evaluation of prognosis after curable resection.

Collagen type III alpha 1 (*COL3A1*) is one of the member of collagen family and mainly expressed in extensible connective tissues including skin and vessels. Mutations in the coding region or the intron region of *COL3A1* gene leads to an arterial disease termed as Ehlers-Danlos syndrome (EDS) and affects patient survival [2-4]. Different genetic variants of *COL3A1* affects the recurrence and prognosis of stroke [5]. The functional variants of *COL3A1* can cause other cardiovascular abnormalities such as intracranial aneurysm [6]. *COL3A1* also plays important roles in cortical development and lamination [7]. The upregulation of *COL3A1* in myofibroblasts contributes to pulmonary fibrosis [8]. *COL3A1* was found to be upregulated in several cancers [9-11], and was revealed to be a candidate diagnostic marker for mesothelioma [11].

The upregulation of *COL3A1* transcription was shown in colorectal cancers comparing with the normal counterparts by microarray gene expression analyses [12-14] and RNA-seq technique [15]. *COL3A1* transcription level was increased from adenoma to carcinoma [16], indicating an involvement of *COL3A1* in carcinogenesis. Interestingly, *COL3A1* was found to be substantially overexpressed in the liver invasion front of the colorectal liver metastases comparing with the tumor center and the normal tissue [17], suggesting a potential role of this gene in the metastasis process. Despite above findings, the functional roles and mechanism of *COL3A1* in tumorigenesis remains elucidative. The relationship of *COL3A1* overexpression with clinicopathological parameters and prognosis requires further explorations.

In current study, we analyzed the mRNA expression level of *COL3A1* in CRC tissue samples by interrogation of publically-available gene expression microarray datasets in Oncomine database. Furthermore, we analyzed the protein expression of COL3A1 protein in CRC using a tissue microarray (TMA) and immunohistochemistry (IHC). The relationship of *COL3A1* expression with the clinic parameters and prognosis outcome of CRC patients were addressed. The protein expression in the plasma of CRC patients was analyzed with enzyme-linked immunosorbent assay (ELISA). *In vitro* and *in vivo* assays were performed to address the function role of COL3A1.

## RESULTS

### *COL3A1* mRNA and protein were significantly upregulated in CRC

**Figure 1:**
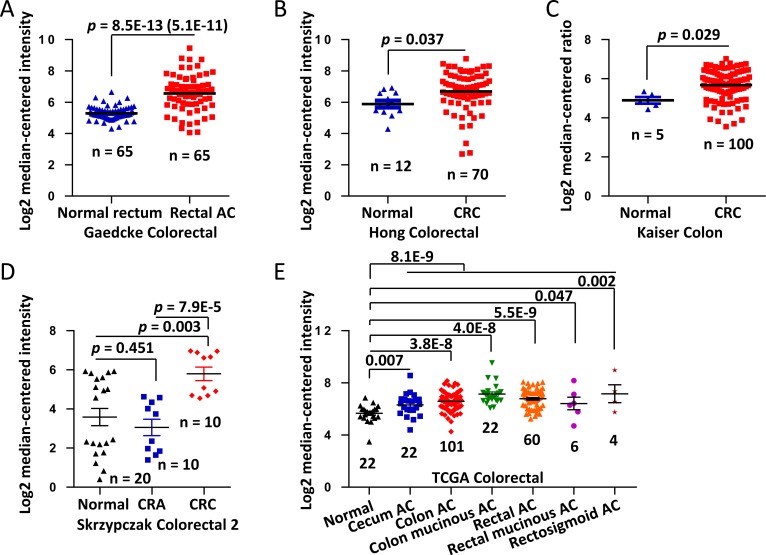
Upregulation of *COL3A1* mRNA in CRC revealed by data-mining of the Oncomine gene expression database **A.** Gene expression of *COL3A1* is upregulated in rectal adenocarcinoma (AC) comparing with the normal rectum tissues revealed using Gaedcke Colorectal dataset from Oncomine DB (https://www.oncomine.org/resource/login.html) [22]. **B.** *COL3A1* gene expression in the normal and CRC tissues by Hong Colorectal dataset [28]. **C.** *COL3A1* expression changes between the normal tissues and cancer tissues of CRC by Kaiser Colon dataset [30]. **D.** *COL3A1* expression analysis in the micro-dissected normal, colorectal adenoma and CRC tissues by Skrzypczak Colorectal 2 dataset [29]. **E.** *COL3A1* expression changes between the normal tissues and different cancer tissues of CRC by TCGA dataset. The n indicates the total cases of different categories. The *p* values are shown above the transverse lines, which are calculated using two-tailed and unpaired Student's *t* test. The *p* value in the blanket is calculated using paired Student's *t* test.

We analyzed the differential expression of *COL3A1* mRNA in colon cancer tissues and the normal counterparts using six microarray gene expression datasets deposited in Oncomine database (DB). *COL3A1* was increased in the cancerous tissues comparing with the normal colonic tissues, as revealed in Gaedcke Colorectal, Hong Colorectal, Kaiser Colon and Skrzypczak Colorectal 2 datasets (Figure 1A-1D). Interestingly, in TCGA Colorectal dataset, *COL3A1* was found to be significantly higher in all types of colon cancers including cecum, colon, rectal, and rectosigmoid cancer (Figure 1E). The total cancer cases were 496 and the normal controls were 148 cases. The results indicated that the overexpression of *COL3A1* in colon cancers is common. *COL3A1* could be a molecular marker of colon cancer.

**Figure 2:**
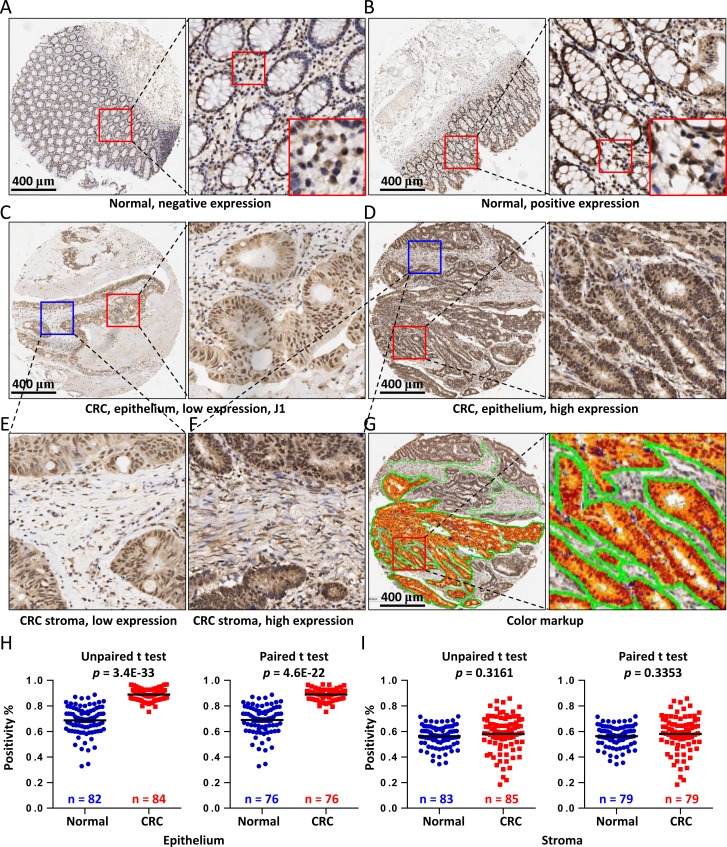
IHC analysis of COL3A1 expression in CRC tissues Negative **A**. or positive **B.** expression of COL3A1 in colonic paraneoplastic mucosa tissues revealed by IHC analysis of a commercial TMA containing 90 cases. The region in the red box was enlarged and displayed to the right side. Scale bar = 400 μm. An area was further enlarged to show the stromal staining of COL3A1. **C.** Low expression of COL3A1 in colorectal adenocarcinoma epithelium. **D.** High expression of COL3A1 in colorectal adenocarcinoma epithelial cells. **E.** Low stromal expression of COL3A1 in the colorectal adenocarcinoma tissue selected from **C.** which was highlighted with blue box. **F.** High stromal expression of COL3A1 in the colorectal adenocarcinoma tissue enlarged from **D.** with blue box. **G.** Pseudo-colored markup of the CRC sample core in **D.** produced by the positive pixel count algorithm in image viewing software Aperio ImageScope v12. **H.** Statistics of epithelial expression of COL3A1 in the colorectal adenocarcinoma and colonic paraneoplastic mucosa tissues based on the positivity values calculated using the positive pixel count algorithm. The left scatterplot displayed the expression of COL3A1 in 82 normal tissues and 84 colorectal cancer (CRC) tissues. The epithelial cells were missing in some other tissues. The *p* values were calculated by unpaired two-sided Student's *t* test and *p* value < 0.05 was considered as statistically significant. The right scatterplot showed only the 76 paired caner-normal tissues. The *p* value is calculated using paired Student's *t* test. **I.** Statistics of stromal expression of COL3A1 in unpaired (left) or paired (right) colorectal adenocarcinoma tissues and colonic paraneoplastic mucosa tissues.

To address the protein expression of COL3A1 in colon cancers, we performed an IHC analysis of COL3A1 expression using a commercially available TMA containing 90 cases of colorectal adenocarcinoma, which 46 cases were completed and 44 were censored (Supplementary Table 1). Negative and positive staining of COL3A1 in the epithelial cells were found in the normal colonic mucosa, while COL3A1 in lamina propria region was low in most cases (Figure 2A and 2B). COL3A1 was expressed in cytoplasm and nucleus and its expression in cancer epithelial and stromal region varied (Figure 1C-1F). To precisely measure the protein expression of COL3A1, we quantified the staining intensity and proportion using the positive pixel count algorithm in Aperio ImageScope software. The negative, weak positive, positive and strong positive pixels were colored as blue, yellow, orange and red, respectively (Figure 2G). In consistent with the mRNA expression, COL3A1 protein was significantly upregulated in cancer epithelial tissues comparing with the normal tissues (unpaired Student's *t* test, *p* = 3.4E-33, *n* = 84, or paired Student's *t* test, *p* = 4.6E-22, *n* = 76) (Figure 2H). However, no significant difference of COL3A1 expression was found between normal and CRC stromal regions (unpaired Student's *t* test, *p* = 0.316, *n* = 85, or paired Student's *t* test, *p* = 0.335, *n* = 79) (Figure 2I).

### Associations of *COL3A1* mRNA and protein expressions with CRC clinicopathological characteristics

We analyzed the relationship of *COL3A1* mRNA level with the clinicopathological characteristics of CRC patients using available Oncomine datasets. *COL3A1* expression was dichotomized into lower-than-median and higher-than-median groups. Expression of *COL3A1* was significantly associated with overall stage (Smith Colorectal [18], *p* = 0.015), T stage (Bittner Colon, *p* = 0.018) and Dukes stage (Bittner Colon, *p* = 0.022; Jorissen Colorectal 3 [19], *p* = 2E-5) (Table 1). A strong correlation between *COL3A1* overexpression and recurrence (Smith Colorectal, *p* = 0.006; Jorissen Colorectal 3, *p* = 0.008) and survival (Smith Colorectal, *p* = 0) was also evident. The analyses of two datasets suggested that expression of *COL3A1* mRNA was associated with age (Vilar Colorectal 2 [20], *p* = 0.014; Jorissen Colorectal 3, borderline *p* = 0.050) (Table 1). There was an evident difference of *COL3A1* expression between male and female patients (Lin Colon 2 [21], *p* = 0.004). High *COL3A1* expression was correlated with smoking status of CRC patients (Bittner Colon, *p* = 0.033).

**Table 1:** Correlation between the mRNA expressions of COL3A1 and clinicopathological variables in patients with colorectal cancer revealed by data-mining of Oncomine gene array datasets

Dataset^a^	Clinicopathological parameters		Total cases	*COL3A1*^b^		χ2	*p*
				< M	≥ M		
Bittner Colon (373)	T stage	Tis-T1	52	35	17	10.073	0.018
		T2	103	47	56		
		T3	85	35	50		
		T4	88	47	41		
	Dukes stage	A	49	33	16	7.607	0.022
		B	101	49	52		
		C-D	88	38	50		
	Smoke	Never smoker	179	100	79	4.523	0.033
		Smoker	194	87	107		
Smith Colorectal (177)	Stage	I	24	19	5	10.468	0.015
		II	57	27	30		
		III	57	23	34		
		IV	39	20	19		
	Recurrence	No recurrence	109	62	47	7.502	0.006
		Recurrence	36	11	25		
	Survival	Alive	104	64	40	12.78	0
		Dead	73	25	48		
Jorissen Colorectal 3 (154)	Dukes stage	A	24	4	20	22.850	0.000
		B	54	24	30		
		C	43	30	13		
		D	33	19	14		
	Recurrence	No recurrence	27	20	7	6.641	0.008
		Recurrence	92	40	52		
	Age	20-49 years	16	12	4	9.491	0.050
		50 - 59 years	29	17	12		
		60 - 69 years	43	20	23		
		70 - 79 years	46	21	25		
		80+ years	24	7	17		
Lin Colon 2 (149)	Sex	Male	70	26	44	8.224	0.004
		Female	79	49	30		
Vilar Colorectal 2 (104)	Age	40-59 years	22	16	6	10.650	0.014
		60 - 69 years	42	26	16		
		70 - 79 years	73	29	44		
		80+ years	39	17	22		

a The analysis was performed using datasets from the Oncomine cancer gene expression microarray DB (https://www.oncomine.org/resource/login.html). The numbers in the blankets represent the total cases.

b *COL3A1* mRNA expression was dichotomized into lower-than-median (< M) and higher-than-median (≥ M) groups.

The overexpression of COL3A1 protein in CRC, based on the positivity by IHC and pixel analysis, was significantly associated with grade (χ2 = 6.731, *p* = 0.029), overall stage (χ2 = 8.438, *p* = 0.004) and T stage (χ2 = 6.923, *p* = 0.012) (Table 2). However, stromal expression of COL3A1 had no significant relationship with any clinicopathological parameters (Supplementary Table 2).

**Table 2:** Correlation of COL3A1 protein expression in cancer epithelial cells with clinicopathological variables in CRC patients revealed by TMA-IHC analysis

Clinicopathological factor	Total cases	COL3A1 protein^a^		χ2	*p* value
		L	H		
Gender					
Male	43	25	18	3.06	0.08
Female	41	16	25		
Age					
< 65 years	30	16	14	0.392	0.531
≥ 65 years	52	24	28		
Grade					
I	5	5	0	6.731	0.029
I-II, II	45	18	27		
I-III, II-III, III	34	18	16		
Vascular Invasion Present					
Positive	3	1	2	0.307	1
Negative	34	17	17		
Number of Positive Lymph Nodes					
< 1	46	25	21	1.141	0.285
≥ 1	31	13	18		
T stage					
T1-T2	9	8	1	6.923	0.012
T3-T4	73	31	42		
N stage					
N0	52	28	24	1.386	0.239
N1-N2	32	13	19		
M stage					
M0	81	39	42	0.003	1
M1	2	1	1		
Stage					
1	7	7	0	8.438	0.004
2-4	75	32	43		
Metastasis					
No	82	40	42	0.001	1
Yes	2	1	1		
Tumor volume (cm3)					
< 70	55	25	30	0.49	0.484
≥ 70	28	15	13		

a The epithelial cell-specific expression of COL3A1 was dichotomized into low (L) or high (H) groups based on the positivity values calculated by the positive pixel count algorithm (v9.1) provided by the Aperio ImageScope v12.1.

### Upregulated *COL3A1* mRNA predicts poor prognosis of CRC patients

**Figure 3:**
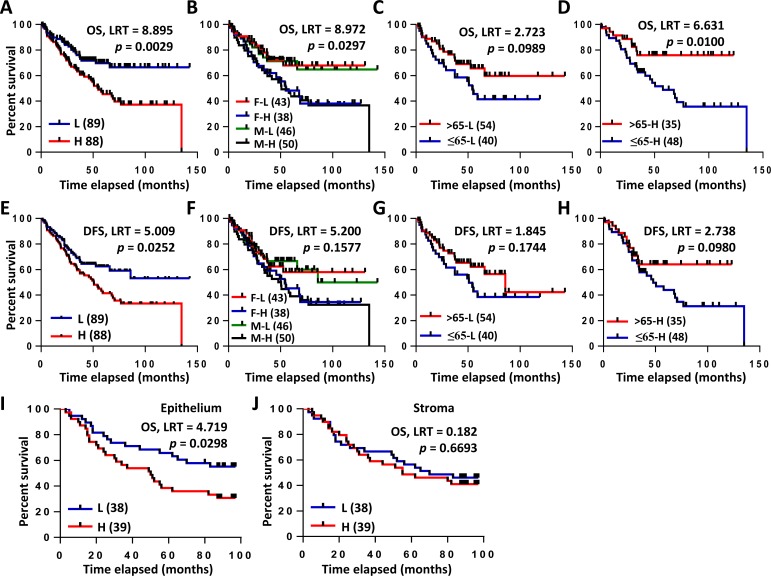
Kaplan-Meier analyses of *COL3A1* mRNA and protein expression in CRC patients **A.**-**F.** Survival analysis of *COL3A1* gene expression in CRC using Smith Colorectal dataset from Oncomine DB [18]. **A.** Overall survival (OS) analysis of low **L.** and high **H.** gene expression of *COL3A1* based on the median value of Log2 median-centered intensity (or ratio). The *p* value was calculated with Log-rank test (LRT) using GraphPad Prism 6. Case numbers were indicated in the brackets. **B.** OS analysis of female/low expression of COL3A1 (F-L), female/high expression (F-H), male/low expression (M-L) and male/high expression (M-H). **C.** OS analysis of young (age > 65) or old (age ≤ 65) with lower-than-median expression of *COL3A1*. **D.** OS analysis of young (age > 65) or old (age ≤ 65) with higher-than-median expression of *COL3A1*. **E.** Disease-free survival (DFS) analysis of *COL3A1* expression as in **A.**. **F.** DFS analysis of different combinations of gender and *COL3A1* expression. **F.** DFS analysis of different combinations of gender and *COL3A1* expression as in **B.**. **G.** DFS analysis of young (age > 65) or old (age ≤ 65) with lower-than-median expression of *COL3A1*. **H.** DFS analysis of young (age > 65) or old (age ≤ 65) with higher-than-median expression of *COL3A1*. **I.** OS analysis of the epithelial expression of COL3A1 protein using the Kaplan-Meier method by the IHC assay. High epithelial expression of COL3A1 was significantly associated with poor outcome of CRC patients. **J.** OS analysis of the stromal expression of COL3A1 protein using the Kaplan-Meier method by the IHC assay.

Next, we proceeded to determine whether the overexpression of *COL3A1* mRNA associates with the prognosis of CRC patients or not. Smith Colorectal dataset in the Oncomine DB contains 177 cases, including 73 completed cases and 104 censored cases. Using Kaplan-Meier method, high expression of *COL3A1* mRNA was found to be significantly associated with poor prognosis of CRC patients in over-all survival (OS) analysis (log-rank test (LRT), *p* = 0.003) (Figure 3A). Since the above analysis indicated that *COL3A1* expression correlated with age and gender, we asked if the combination of these parameters associated with outcome. Either in female or male patients, high expression of *COL3A1* correlated with worse survival (LRT, *p* = 0.030) (Figure 3B). Although younger (age ≤ 65) or older (age > 65) patients had no significant difference in survival when *COL3A1* was low (LRT, *p* = 0.099) (Figure 3C), the survival of older patients with high *COL3A1* was considerably worse than younger patients with high *COL3A1* (LRT, *p* = 0.010) (Figure 3D). An increased level of *COL3A1* mRNA also predicted worse recurrence-free outcome of CRC patients (LRT, *p* = 0.025) (Figure 3E). Different combinations of gender or age with *COL3A1* expression had no significant difference in their disease-free survival (DFS) (Figure 3F-3H).

Univariate and multivariate Cox regression analyses were performed to evaluate the association of *COL3A1* and clinic factors with prognostic hazards. In univariate Cox analysis, *COL3A1* (*p* = 0.004), grade (*p* = 0.004), recurrence status (*p* = 0.000) and stage (*p* = 0.000) were found to have an adverse impact on OS (Table 3). Whereas increased *COL3A1* (*p* = 0.027), high grade (*p* = 0.003), recurrence status (*p* = 0.000) and high stage (*p* = 0.000) were significantly associated with inferior DFS (Table 3). In multivariate analysis, both recurrence status and stage were significant predictors of OS (recurrence: Hazard ratio (HR) = 4.084, *p* = 0.000; stage: HR = 1.554, *p* = 0.033) and DFS (recurrence: HR = 8.276, *p* = 0.000; stage: HR = 1.536, *p* = 0.029) (Table 3).

**Table 3:** Univariate and multivariate Cox regression analyses of COL3A1 mRNA expression and clinical variables for overall and disease-free survival of CRC patients^a^

Survival	Methods	Variables	β^b^	SE (β)^c^	Wald χ2	*p*	Hazard ratio	95% CI^d^	
								Low	Upper
Overall-survival									
	Univariate Cox regression								
		Age	0.006	0.009	0.472	0.492	1.006	0.988	1.025
		COL3A1	0.720	0.247	8.517	0.004	2.055	1.267	3.333
		Grade	0.695	0.241	8.311	0.004	2.004	1.249	3.215
		Recurrence	1.569	0.308	25.958	0.000	4.801	2.625	8.778
		Sex	0.100	0.237	0.176	0.675	1.105	0.694	1.759
		Stage	1.049	0.154	46.482	0.000	2.854	2.111	3.859
	Multivariate Cox regression								
		Recurrence	1.407	0.315	20.010	0.000	4.084	2.205	7.565
		Stage	0.441	0.207	4.531	0.033	1.554	1.036	2.333
Disease-Free survival									
	Univariate Cox regression								
		Age	0.003	0.008	0.116	0.733	1.003	0.986	1.020
		COL3A1	0.500	0.226	4.903	0.027	1.648	1.059	2.565
		Grade	0.692	0.230	9.062	0.003	1.997	1.273	3.133
		Recurrence	2.272	0.293	60.110	0.000	9.703	5.463	17.235
		Sex	0.051	0.222	0.052	0.819	1.052	0.681	1.626
		Stage	0.985	0.141	48.820	0.000	2.677	2.031	3.528
	Multivariate Cox regression								
		Recurrence	2.113	0.299	49.839	0.000	8.276	4.603	14.882
		Stage	0.429	0.197	4.742	0.029	1.536	1.044	2.259

a The analysis was performed using the Smith Colorectal dataset from the Oncomine cancer gene expression microarray DB (https://www.oncomine.org/resource/login.html).

b β, regression coefficient.

c SE, standard error.

d CI, confidence interval.

### Epithelial but not stromal expression of COL3A1 protein associated with poor outcome of CRC

Different from gene expression microarray, protein IHC provides more information of the protein expression location and the abundance difference between locations. Interestingly, the increased COL3A1 protein in cancer epithelial region was significantly associated with poor OS revealed by the Kaplan-Meier analysis (LRT, *p* = 0.03) (Figure 3I). However, cancer stromal expression of COL3A1 had no significant relationship with OS (LRT, *p* = 0.669) (Figure 3J).

Univariate Cox regression analysis revealed that COL3A1 (epithelial) (*p* = 0.030), T stage (*p* = 0.002), N stage (*p* = 0.000), M stage (*p* = 0.000), stage (*p* = 0.000) and metastasis status (*p* = 0.002) were significantly associated with inferior OS (Table 4). While multivariate analysis indicated that T (HR = 2.173, *p* = 0.001), N (HR = 1.233, *p* = 0.000) and M stage (HR = 37.056, *p* = 0.001) were independent hazard factors of CRC patients (Table 4).

**Table 4:** Univariate and multivariate Cox regression analyses of COL3A1 protein expression in cancer epithelial cells and clinical variables for overall survival of CRC patients

Methods	Variables	β^a^	SE (β)^b^	Wald χ2	*p*	Hazard ratio	95% CI^c^	
							Low	Upper
Univariate Cox regression								
	Age	0.010	0.011	0.910	0.340	1.010	0.989	1.032
	Gender	−0.323	0.289	1.245	0.264	0.724	0.411	1.276
	COL3A1 (epithelial)	0.719	0.332	4.697	0.030	2.053	1.071	3.935
	Grade	0.223	0.150	2.228	0.136	1.250	0.932	1.677
	T	0.636	0.207	9.442	0.002	1.889	1.259	2.834
	N	0.428	0.098	19.094	0.000	1.534	1.266	1.859
	M	2.350	0.626	14.099	0.000	10.484	3.075	35.747
	Stage	0.271	0.068	15.794	0.000	1.311	1.147	1.499
	Metastasis	2.315	0.757	9.341	0.002	10.123	2.294	44.664
	COL3A1 (stromal)	0.128	0.302	0.181	0.671	1.137	0.629	2.055
	Tumor volume	0.000	0.000	2.002	0.157	1.000	1.000	1.001
Multivariate Cox regression								
	T	0.776	0.244	10.124	0.001	2.173	1.347	3.506
	N	0.426	0.111	14.815	0.000	1.532	1.233	1.903
	M	3.612	1.038	12.101	0.001	37.056	4.841	283.663

a β, regression coefficient.

b SE, standard error.

c CI, confidence interval.

### COL3A1 is a plasma marker of CRC

**Figure 4:**
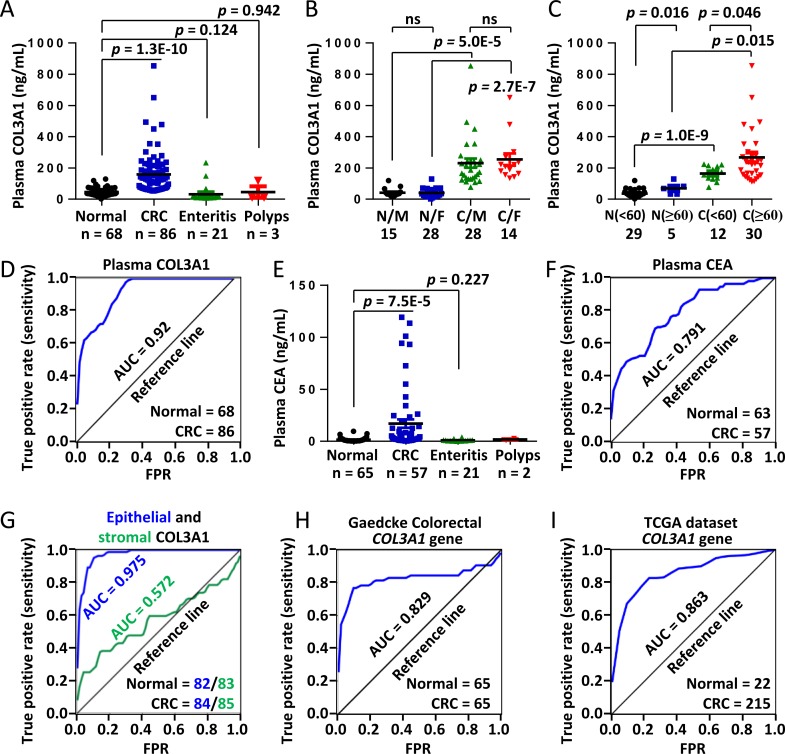
Elevated plasma COL3A1 protein is a potential diagnostic marker of CRC **A.** The plasma expressions of COL3A1 in 68 healthy individuals, 86 CRCs, 21 enteritis and 3 polyps patients were measured using a sandwich ELISA method. The concentration of COL3A1 was calculated comparing with the standard curve. The *p* values were calculated using two-sided Student's *t* test and a *p* value < 0.05 was considered as statistically significant. The case numbers were indicated below each categories. **B.** Comparison of plasma COL3A1 between normal/male (N/M), normal/female (N/F), CRC/male (C/M) and CRC/female (C/F). ns, non-significant. **C.** Comparison of plasma COL3A1 between normal/(age < 60), normal/(age ≥ 60), CRC/(age < 60) and CRC/(age ≥ 60). **D.** ROC curve analysis of plasma COL3A1 in the CRC patients (*n* = 86) and the healthy controls (*n* = 68). The best diagnostic sensitivity and specificity of COL3A1 were 98.8% and 69.1%, respectively, at a plasma concentration of 54.23 ng/mL. AUC, area under curve. FPR, false positive rate. **E.** ELISA analysis of carcino-embryonic antigen (CEA) in the plasma samples. **F.** ROC analysis of CEA in the same set of plasma samples. The best diagnostic sensitivity and specificity of CEA were 70.2% and 73.0%, respectively, at a plasma concentration of 1.68 ng/mL. **G.** ROC analysis of the epithelial and stromal expression of COL3A1 in CRC tissues using IHC results. The best sensitivity and specificity of epithelial COL3A1 were 95.2% and 91.1%, respectively. The best sensitivity and specificity of stromal COL3A1 were 38.1% and 86.1%, respectively. **H.** ROC analysis of the mRNA expression of *COL3A1* in CRC tissues using Gaedcke Colorectal dataset in Oncomine database [22]. The best sensitivity and specificity were 76.9% and 92.3%, respectively. **I.** ROC analysis of the mRNA expression of *COL3A1* in CRC tissues using TCGA Colorectal dataset in Oncomine database. The best sensitivity and specificity were 82.8% and 81.8%, respectively.

To address the diagnostic significance of circulated COL3A1 as a protein marker of CRC, we analyzed the plasma concentration of COL3A1 using ELISA method. The sample information and ELISA results were deposited in Supplementary Table 3. Plasma COL3A1 of the CRC patients (*n* = 86), but not enteritis (*n* = 21) and polyps (*n* = 3) patients, was significantly higher than that of the healthy donors (*n* = 68) (unpaired Student's *t* test, *p* = 1.3E-10) (Figure 4A). The average concentration of plasma COL3A1 in the CRC group (134.05 ng/mL) was 5.4 fold higher than that in the healthy group (24.68 ng/mL). The average COL3A1 level (21.83 ng/mL) in the enteritis group was similar to the normal group, after excluding an extreme value. No matter what sex the patient was, plasma COL3A1 was significant higher in CRC than healthy donors (Figure 4B). While plasma COL3A1 had no difference between male and female in normal or CRC patients. In younger (age < 60) or older (age ≥ 60) individuals, plasma COL3A1 was significantly higher in the cancer group than the healthy group (Figure 4C). Interestingly, plasma COL3A1 was notably increased in older peoples in either healthy (*p* = 0.016) or cancer individuals (*p* = 0.046). Receiver operating characteristic (ROC) curve analysis revealed that plasma COL3A1 had an area under curve (AUC) of 0.92, indicating a good prediction power for CRC (Figure 4D). The best diagnostic sensitivity and specificity of plasma COL3A1 were 98.8% and 69.1%, respectively, at a plasma concentration of 54.23 ng/mL. For comparison, we measured the plasma concentration of carcino-embryonic antigen (CEA), a typical tumor marker currently used for diagnosis. CEA was low in the normal (average = 1.49 ng/mL), enteritis (average = 0.95 ng/mL) and polyps group (average = 1.74 ng/mL), but was considerably increased by 17 fold in the CRC group (average = 17.08 ng/mL) (Figure 4E). The AUC of plasma CEA was 0.791, with the best diagnostic sensitivity and specificity of CEA were 70.2% and 73.0%, respectively, at a plasma concentration of 1.68 ng/mL (Figure 4F). The results demonstrated that plasma COL3A1 was comparable to CEA in CRC diagnosis.

We also analyzed the predictive power of COL3A1 expression in TMA of CRC. The epithelial expression of COL3A1 had the best AUC of 0.975, and the best sensitivity and specificity were 95.2% and 91.1%, respectively (Figure 4G). However, the stromal expression of COL3A1 had no clinic prediction value with an AUC of 0.573 (Figure 4G). We further evaluated the capability of *COL3A1* mRNA expression to distinguish CRC patients from healthy people. ROC analysis using Gaedcke Colorectal dataset in Oncomine database [22] revealed that *COL3A1* gene expression had an AUC of 0.829 and the best sensitivity and specificity were 76.9% and 92.3%, respectively (Figure 4H). In TCGA Colorectal dataset, the AUC of COL3A1 was 0.863 by ROC analysis and the best sensitivity and specificity were 82.8% and 81.8%, respectively (Figure 4I).

### COL3A1 promotes CRC cell proliferation by stimulating PI3K-AKT signaling

**Figure 5:**
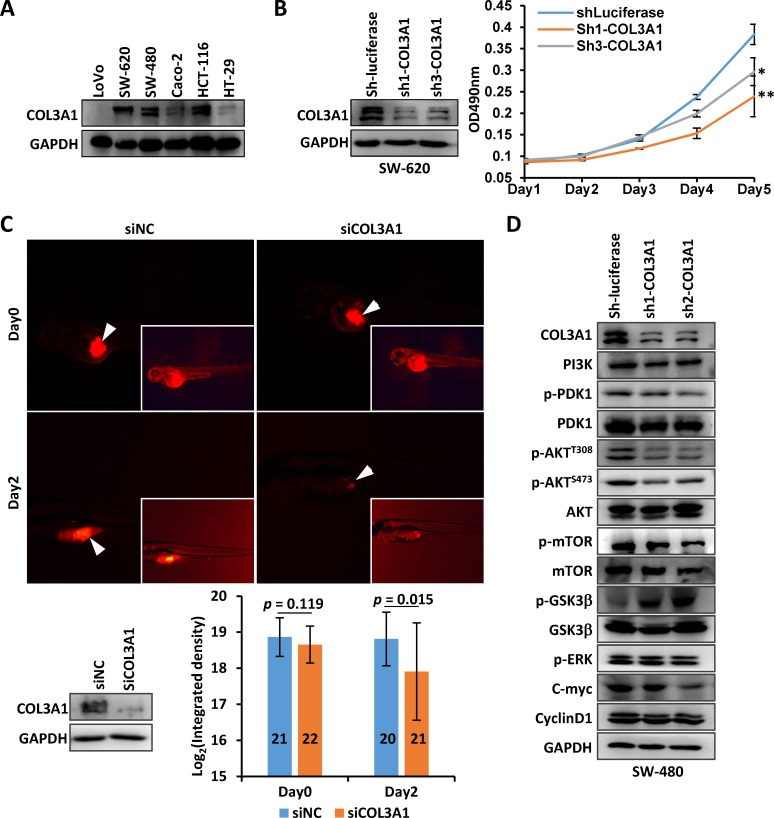
Silencing of COL3A1 promotes CRC cells proliferation **A.** COL3A1 protein expression was measured in CRC cell lines. **B.** *COL3A1* was silenced in SW-620 cells with shRNAs and the growth of SW-620 was analyzed using MTT method. *p* value < 0.05 was indicated with *, while *p* < 0.01 was indicated with **. **C.** Zebra fish cancer xenograft assay of SW-620 cells with silenced *COL3A1* by a siRNA. The photos at the same time point were acquired with the same exposure time setting. The inserted pictures were modified photos with increased brightness to show the fishes. The arrowheads indicated the positions of amplified CRC cells. The silencing of *COL3A1* was verified by Western blot (bottom left). The integrated density of fluorescence of amplified cells were calculated with Image J (lower right). The numbers of fishes were shown within the column. **D.** Western blot analyses of several signaling molecules by *COL3A1*-knockdown in CRC cells.

It was intriguing that COL3A1 protein localized on nucleus of cancer epithelial cells, in addition to the cytoplasm and extracellular space (Figure 2). This observation has never been documented in previous studies. It raised a hypothesis that COL3A1, a typical extracellular matrix (ECM) protein, might function as a signaling molecule within nucleus. We first analyzed the expression of COL3A1 in CRC cell lines. As shown in Figure 5A, COL3A1 was abundantly expressed in SW-620, SW-480 and HCT-116 cells. Endogenous *COL3A1* was stably silenced in SW-620 using short hairpin RNAs (shRNAs), and cell proliferation was measured using the methylthiazolyldiphenyl-tetrazolium bromide (MTT) method. The proliferation of SW-620 cells was suppressed by *COL3A1* knockdown (Figure 5B). We further analyzed *COL3A1* silencing *in vivo* using a zebra fish xenograft model, which has been used in cancer proliferation, cell invasion and metastasis analyses [23]. Endogenous *COL3A1* was silenced with a small interference RNA (siRNA) and the cells were treated with a fluorescent dye before inoculated into the fish yolk. The fluorescence in the yolk was measured to reflect the proliferation. As displayed in Figure 5C, *COL3A1* knockdown notably reduced cancer cell proliferation 2 days post-inoculation. Next we asked what signaling pathway(s) that *COL3A1* might stimulate. After *COL3A1* silencing, p-AKT^T308^, p-AKT^S473^ and p-mTOR^S2448^ were downregulated while p-GSK-3α/β^S21/9^ was upregulated, indicating a phosphorylation cascade of PI3K/AKT pathway (Figure 5D). Meanwhile, c-Myc was notably suppressed while p-ERK and cyclin D1 were less affected. These observation suggested loss of *COL3A1* inhibited PI3K/AKT and WNT signaling in CRC cells.

## DISCUSSION

In this study, by analyzing Oncomine datasets, we found that *COL3A1* expression was significantly upregulated in colon cancers (total 654 cases) comparing with normal controls (178 samples). Furthermore, *COL3A1* was increased in all six subtype of colorectal cancers. These observation were in line with previous findings that *COL3A1* gene was upregulated in colon cancers [12-15]. We further revealed that *COL3A1* expression was significantly associated with clinic parameters of CRC in different cohorts, like age, sex, stage, T stage, Dukes stage, tobacco consumption, recurrence and survival status. Whereas upregulation of COL3A1 protein was found to be associated with grade, stage and T stage of CRC patient. These observation suggested that COL3A1 was a potential molecular marker of CRC.

A previous report indicates that *COL3A1* gene had a prognostic implication in brain tumor [24]. However, its prognostic significance in CRC remains undetermined. Here we revealed that upregulation of *COL3A1* mRNA was significantly associated with poor outcome of CRC patients in OS or DFS analyses using Oncomine datasets. Kaplan-Meier survival analysis revealed that increased COL3A1 protein in cancer epithelial cells predicted worse prognosis. These results suggested that *COL3A1* mRNA and protein could be prognosis indicators of CRC. Univariate but not multivariate Cox regression analyses demonstrated that *COL3A1* mRNA and epithelial-specific COL3A1 protein expression were significant hazard factors for CRC. While multivariate analyses revealed that stage and recurrence (mRNA datasets) or T, N and M stage (the protein analysis) were independent risk factors. These results suggested that *COL3A1* might not be an independent (or major) hazard factor for CRC patient survival.

As a soluble extracellular protein, COL3A1 was expected to be released into circulation system. Here we analyzed the plasma COL3A1 of CRC patients using the ELISA method. Comparing with the healthy peoples, the CRC patients have significant higher concentration of circulating COL3A1. Interestingly, plasma COL3A1 had a good prediction power with an AUC of 0.92, a best sensitivity of 98.8% at a specificity of 69.1%. Plasma CEA had a poor prediction power but a better specificity than plasma COL3A1. Tissue staining of epithelial-specific COL3A1 expression had a superior prediction value of AUC 0.975 and the highest sensitivity/specificity of 95.2%/91.1%, which was comparable to spondin-2, another molecular marker of colon cancer [25]. However, stromal COL3A1 had less power to discriminate CRC from healthy individuals (AUC = 0.572). *COL3A1* mRNA could also be used as a molecular diagnostic marker. However, *COL3A1* mRNA expression did not performed as good as the epithelial COL3A1. After all, mRNAs were extracted from tissues mixed with heterogeneous type of cells, while IHC analysis had the advantage to focus on cancer-specific cells. Thus, plasma COL3A1 represents a convenient molecular marker for quick and non-invasive diagnosis, whereas the IHC staining of epithelial COL3A1 could be useful for post-surgery outcome evaluation.

COL3A1 has found to be increased in the stroma of other type of cancers [26]. We also previously identified COL3A1 in the secretome of colon cancer-associated fibroblast (CAF) [27]. However, by IHC analysis of CRC tissues, COL3A1 was unexpectedly found to be expressed in the nuclei of epithelial cells. This observation indicated that COL3A1 might be function as a nuclear protein in addition to be a secreted extracellular matrix protein. We found that COL3A1 promotes CRC cell proliferation by stimulating PI3K-AKT signaling, suggesting COL3A1 could be a potential therapeutic target.

In summary, our study demonstrated that COL3A1 mRNA and protein were upregulated in CRC which were associated with clinicopathological factors and poor survival, and COL3A1 was also increased in plasma of CRC patient. COL3A1 could be a potential diagnostic biomarker of colon cancer.

## MATERIALS AND METHODS

### Cell lines and cell culture

CRC cell lines LoVo, SW-480, SW-620, Caco-2 and HCT-116 were purchased from the Cell Bank of Shanghai Institutes for Biological Sciences, China. SW480 and SW-620 cells were cultured in DMEM supplemented with 10% FBS. Caco-2 cells were cultured in DMEM supplemented with 20% FBS. LoVo cells were cultured in RPMI 1640 supplied with 10% FBS. HCT-116 and HT-29 cells were cultivated with McCoy's 5A medium (Gibco Life Technologies, Shanghai, China)/10% FBS. All media were supplemented with 1% penicillin and 1% streptomycin (Hyclone Laboratories, China). Cells were maintained in a humidified incubator at 37°C and 5% CO_2_.

### Data-mining of oncomine gene expression microarray datasets

We analyzed the mRNA expression of *COL3A1* in GC using the Oncomine cancer gene expression microarray database (DB) (https://www.oncomine.org/resource/login.html). The data retrieve and analysis method has been previously described in detail [25]. The Smith Colorectal dataset contains 177 cases, including 73 completed cases and 104 censored cases [18]. Other datasets used in current study were Gaedcke Colorectal [22], Hong Colorectal [28], Skrzypczak Colorectal 2 [29] and Kaiser Colon dataset [30]. In each datasets, the *COL3A1* expression was dichotomized into lower-than-median and higher-than-median expression group based on the Log2 median-centered intensity of *COL3A1* mRNA.

### Antibodies

Antibodies of COL3A1 (13548-1-AP), cyclin D1 (60186-1-Ig) and PI3K (20583-1-AP) were purchased from Proteintech (Wuhan, Hubei, China). GAPDH antibody (CW0100) was bought from CWBIO, Beijing, China. Other antibodies were purchased from Cell Signaling Technology (CST): PDK1 (#3062), p-PDK1S241 (#3061), p-AktS473 (D9E, #4060), p-AktT308 (244F9, 4056), p-p70S6KT389 (108D2, #9234), p70 S6 Kinase (49D7, #2708), p-mTORS2448 (#2971), mTOR (#2972), p-GSK-3α/βS21/9 (D17D2, #8566) and GSK-3α/β (D75D3,#5676).

### RNA interference (RNAi)

RNAi was performed by transfection of CRC cells with a siRNA against COL3A1. The siRNA for *COL3A1* and a negative control (siNC) were purchased from Shanghai GenePharma Co., Ltd. The siRNA sequence used for COL3A1 was: GAGACUACCUAUUGUAGAUdTdT. Transfection was performed using Lipofectamine 2000 (Life Technologies - Invitrogen) with the aid of OPTI-MEM reduced serum media (Life Technologies - Invitrogen) according to the manufacturer's instructions and the methods as described previously [27, 31, 32].

### shRNA lentivirus production

The shRNA lentiviral plasmid (pLKO.1) was obtained from The RNAi Consortium. The core sequences of shRNAs against *COL3A1* were as following: sh1-COL3A1, CCGTTCTCTGCGATGACATAA; sh2-COL3A1, ACACCGATGAGATTATGACTT; sh3-COL3A1, AGCTACGGCAATCCTGAACTT. A shRNA against luciferase was used as a control: GTGCGCTGCTGGTGCCAAC. Lentivirus was produced using HEK293FT cells with the second-generation packaging system psPAX2 (Addgene plasmid 12260) and pMD2.G (Addgene plasmid12259) as described previously [31, 32].

### Cellular protein extraction, Western blot and growth curve assay

Protein extraction and Western blot were performed as described previously [31]. The proliferation of CRC cells was measured using MTT (Sigma-Aldrich, Shanghai, China) method in 96-well plate. Each time point had at least five replicates. The MTT transformed crystals were dissolved in DMSO. The absorbance at 490 nm was measured using an Epoch microplate spectrophotometer (BioTek).

### Zebrafish xenograft cancer model

The maintenance of Zebrafish and cancer cell inoculation were performed according to the methods established as described previously [32]. The difference was that the CRC cells were stained with 6 μM fluorescent CellTracker CM-Dil (Invitrogen) at 37°C for 4 min, followed by a further incubation at 4°C for 15 min. The fishes were incubated for two days and photographed using a LEICA M205 FA stereo fluorescence microscope with the same exposure time for both the control and knockdown group. The magnification was 140 ×. The integrated density of fluorescence was measured using Image J 1.45s.

### IHC analysis

IHC was performed with COL3A1 antibody at a 1:800 dilution using a commercial TMA (catalog no. HCol-Ade180Sur-04, Shanghai Outdo Biotech, China) according to an established protocol of Outdo Biotech as previously described [25, 27, 32]. The TMA consists of 90 pairs of colorectal adenocarcinoma tissues and the matched normal mucosa counterparts, including 49 completed cases and 41 censored cases. The surgical time was from July 2006 to May 2007 and the follow-up information was available from Nov. 2006 to Aug. 2014. The survival time was from 3 to 85 months with a median of 56 months and 46 patients had died. The clinicopathological features of the GC patients were listed in Supplementary Table 1. After stained sequentially with COL3A1 antibody and a second antibody, the TMA was incubated with a substrate 3,3′-diaminobenzidine (DAB) for color developing and the nuclei were counterstained with hematoxylin. The TMA was then scanned using Scanscope XT (Aperio Technologies). The interesting regions in each sample core, such as cancer epithelial cells, cancer stromal region, epithelial cells or lamina propria region in paired normal mucosa tissues, were selected and labeled at different layers with the annotation function in Aperio ImageScope (v12.1) software. While the unwanted areas within the selected regions were excluded with the negative labeling tool. The staining intensity and percentage of selected sample regions were quantified with default settings using the positive pixel count algorithm (v9.1) provided by the Aperio ImageScope. The pixels were recognized as negative, weak positive, positive and strong positive pixels based on the default color and intensity thresholds. A color markup was generated by the analysis, which was used for checking and verification of each selection and coloration. The positivity was calculated as that the number of positive pixels was divided by the total number of all negative and positive pixels in the analyzed region. The expression was dichotomized into low and high by the median positivity value.

### ELISA

Clinic plasma samples from health individuals and CRC patients were collected from the First Affiliated Hospital of Soochow University, Jiangsu, China, and the study was approved by the Clinical Research Ethics Committee of the First Affiliated Hospital of Soochow University. The sample information was listed in Supplementary Table 3. The plasma samples were stored in small aliquots at −80°C before use. ELISA was performed using a sandwich enzyme immunoassay method with a pre-coated 96-well strip plate from an ELISA kit for COL3A1 (Cloud-Clone Corp product, purchased from Wuhao, Inc., Shanghai, China) according to the manufacturer's instructions. The optimal dilution of the plasma samples was determined by serial dilution, which was comparing with the results of diluted standard provided by the ELISA kit. The plasma samples were diluted 20 folds with PBS (pH 7.2) before analyzing. The reaction was developed using the tetramethyl benzidine (TMB) substrate and the absorbance was measured using a microplate reader at a wave length of 450 nm. The concentrations of COL3A1 in each plasma sample were calculated using the standard curve.

### Statistical analysis

The significance was calculated using two-tailed Student's *t* test and a *p* value < 0.05 was considered as statistically significant. The correlation between *COL3A1* expression and the clinicopathological parameters were evaluated by χ2 test using PASW Statistics 18. Kaplan-Meier survival curves were calculated using the log-rank test with GraphPad Prism 6.01 and a *p* value < 0.05 was considered as statistically significant. Univariate or multivariate survival analyses using Cox proportional hazard regression model were performed using PASW Statistics 18. The ROC curve analysis was performed using GraphPad Prism 6.0.1 and the optimal concentration of COL3A1 was determined at the maximal sensitivity and specificity.

### SUPPLEMENTARY MATERIAL TABLES



## Abbreviations

AUC: area under curve
CAF: cancer-associated fibroblast
CEA: carcino-embryonic antigen
COL3A1: collagen alpha-1(III)
CRC: colorectal cancer
DAB: 3,3′-diaminobenzidine
DB: database
DFS: Disease-free survival
ECM: extracellular matrix
EDS: Ehlers-Danlos syndrome
ELISA: enzyme-linked immunosorbent assay
HR: hazard ratio
IHC: immunohistochemistry
LRT: log-rank test
OS: over-all survival
RNAi: RNA interference
ROC: receiver operating characteristic
shRNA: short hairpin RNAs
siRNA: small interference RNA
TMA: tissue microarray
TMB: tetramethyl benzidine

## References

[1] World Health Organization - International Agency for Research on Cancer. GLOBOCAN Cancer Fact Sheets http://globocan.iarc.fr/Pages/fact_sheets_cancer.aspx?cancer=colorectal.

[2] Franzon VL, Gibson MA, Hatzinikolas G, Woollatt E, Sutherland GR, Cleary EG (1999). Molecular cloning of a novel human PAPS synthetase which is differentially expressed in metastatic and non-metastatic colon carcinoma cells. Int J Biochem Cell Biol.

[3] Balakrishnan L, Nirujogi RS, Ahmad S, Bhattacharjee M, Manda SS, Renuse S, Kelkar DS, Subbannayya Y, Raju R, Goel R, Thomas JK, Kaur N, Dhillon M, Tankala SG (2014). Proteomic analysis of human osteoarthritis synovial fluid. Clin Proteomics.

[4] Manda R, Kohno T, Matsuno Y, Takenoshita S, Kuwano H, Yokota J (1999). Identification of genes (SPON2 and C20orf2) differentially expressed between cancerous and noncancerous lung cells by mRNA differential display. Genomics.

[5] Lv W, Lin Y, Song W, Sun K, Yu H, Zhang Y, Zhang C, Li L, Suo M, Hui R, Chen J (2014). Variants of COL3A1 are associated with the risk of stroke recurrence and prognosis in the Chinese population: a prospective study. J Mol Neurosci.

[6] Whitaker HC, Shiong LL, Kay JD, Gronberg H, Warren AY, Seipel A, Wiklund F, Thomas B, Wiklund P, Miller JL, Menon S, Ramos-Montoya A, Vowler SL (2014). N-acetyl-L-aspartyl-L-glutamate peptidase-like 2 is overexpressed in cancer and promotes a pro-migratory and pro-metastatic phenotype. Oncogene.

[7] Barbieri CE (2013). Evolution of novel biomarkers for detection of prostate cancer. J Urol.

[8] Kim JW, Kim ST, Turner AR, Young T, Smith S, Liu W, Lindberg J, Egevad L, Gronberg H, Isaacs WB, Xu J (2012). Identification of new differentially methylated genes that have potential functional consequences in prostate cancer. PloS one.

[9] Turashvili G, Bouchal J, Baumforth K, Wei W, Dziechciarkova M, Ehrmann J, Klein J, Fridman E, Skarda J, Srovnal J, Hajduch M, Murray P, Kolar Z (2007). Novel markers for differentiation of lobular and ductal invasive breast carcinomas by laser microdissection and microarray analysis. BMC cancer.

[10] Tilman G, Mattiussi M, Brasseur F, van Baren N, Decottignies A (2007). Human periostin gene expression in normal tissues, tumors and melanoma: evidences for periostin production by both stromal and melanoma cells. Mol Cancer.

[11] Rieder D, Ploner C, Krogsdam AM, Stocker G, Fischer M, Scheideler M, Dani C, Amri EZ, Muller WG, McNally JG, Trajanoski Z (2014). Co-expressed genes prepositioned in spatial neighborhoods stochastically associate with SC35 speckles and RNA polymerase II factories. Cell Mol Life Sci.

[12] Liao CH, Yeh SC, Huang YH, Chen RN, Tsai MM, Chen WJ, Chi HC, Tai PJ, Liao CJ, Wu SM, Cheng WL, Pai LM, Lin KH (2010). Positive regulation of spondin 2 by thyroid hormone is associated with cell migration and invasion. Endocr Relat Cancer.

[13] Bourdonnay E, Morzadec C, Sparfel L, Galibert MD, Jouneau S, Martin-Chouly C, Fardel O, Vernhet L (2009). Global effects of inorganic arsenic on gene expression profile in human macrophages. Mol Immunol.

[14] Cohen CD, Lindenmeyer MT, Eichinger F, Hahn A, Seifert M, Moll AG, Schmid H, Kiss E, Grone E, Grone HJ, Kretzler M, Werner T, Nelson PJ (2008). Improved elucidation of biological processes linked to diabetic nephropathy by single probe-based microarray data analysis. PloS one.

[15] Simon I, Liu Y, Krall KL, Urban N, Wolfert RL, Kim NW, McIntosh MW (2007). Evaluation of the novel serum markers B7-H4, Spondin 2, and DcR3 for diagnosis and early detection of ovarian cancer. Gynecol Oncol.

[16] Paper W, Kroeber M, Heersink S, Stephan DA, Fuchshofer R, Russell P, Tamm ER (2008). Elevated amounts of myocilin in the aqueous humor of transgenic mice cause significant changes in ocular gene expression. Exp Eye Res.

[17] Pham VC, Henzel WJ, Lill JR (2005). Rapid on-membrane proteolytic cleavage for Edman sequencing and mass spectrometric identification of proteins. Electrophoresis.

[18] Bingham SA, Day NE, Luben R, Ferrari P, Slimani N, Norat T, Clavel-Chapelon F, Kesse E, Nieters A, Boeing H, Tjonneland A, Overvad K, Martinez C (2003). Dietary fibre in food and protection against colorectal cancer in the European Prospective Investigation into Cancer and Nutrition (EPIC): an observational study. Lancet.

[19] Day LW, Velayos F (2014). Colorectal cancer of the elderly. Curr Treat Options Gastroenterol.

[20] Vilar E, Bartnik CM, Stenzel SL, Raskin L, Ahn J, Moreno V, Mukherjee B, Iniesta MD, Morgan MA, Rennert G, Gruber SB (2011). MRE11 deficiency increases sensitivity to poly(ADP-ribose) polymerase inhibition in microsatellite unstable colorectal cancers. Cancer research.

[21] Guffey CR, Fan D, Singh UP, Murphy EA (2013). Linking obesity to colorectal cancer: recent insights into plausible biological mechanisms. Curr Opin Clin Nutr Metab Care.

[22] Gaedcke J, Grade M, Jung K, Camps J, Jo P, Emons G, Gehoff A, Sax U, Schirmer M, Becker H, Beissbarth T, Ried T, Ghadimi BM (2010). Mutated KRAS results in overexpression of DUSP4, a MAP-kinase phosphatase, and SMYD3, a histone methyltransferase, in rectal carcinomas. Genes Chromosomes Cancer.

[23] Cock-Rada AM, Medjkane S, Janski N, Yousfi N, Perichon M, Chaussepied M, Chluba J, Langsley G, Weitzman JB (2012). SMYD3 promotes cancer invasion by epigenetic upregulation of the metalloproteinase MMP-9. Cancer research.

[24] Hao JM, Chen JZ, Sui HM, Si-Ma XQ, Li GQ, Liu C, Li JL, Ding YQ, Li JM (2010). A five-gene signature as a potential predictor of metastasis and survival in colorectal cancer. J Pathol.

[25] Zhang Q, Wang XQ, Wang J, Cui SJ, Lou XM, Yan B, Qiao J, Jiang YH, Zhang LJ, Yang PY, Liu F (2015). Upregulation of spondin-2 predicts poor survival of colorectal carcinoma patients. Oncotarget.

[26] Kauppila S, Stenback F, Risteli J, Jukkola A, Risteli L (1998). Aberrant type I and type III collagen gene expression in human breast cancer *in vivo*. J Pathol.

[27] Chen SX, Xu XE, Wang XQ, Cui SJ, Xu LL, Jiang YH, Zhang Y, Yan HB, Zhang Q, Qiao J, Yang PY, Liu F (2014). Identification of colonic fibroblast secretomes reveals secretory factors regulating colon cancer cell proliferation. J Proteomics.

[28] Edwards S, Campbell C, Flohr P, Shipley J, Giddings I, Te-Poele R, Dodson A, Foster C, Clark J, Jhavar S, Kovacs G, Cooper CS (2005). Expression analysis onto microarrays of randomly selected cDNA clones highlights HOXB13 as a marker of human prostate cancer. British journal of cancer.

[29] Skrzypczak M, Goryca K, Rubel T, Paziewska A, Mikula M, Jarosz D, Pachlewski J, Oledzki J, Ostrowski J (2010). Modeling oncogenic signaling in colon tumors by multidirectional analyses of microarray data directed for maximization of analytical reliability. PloS one.

[30] Trueb B, Neuhauss SC, Baertschi S, Rieckmann T, Schild C, Taeschler S (2005). Fish possess multiple copies of fgfrl1, the gene for a novel FGF receptor. Biochim Biophys Acta.

[31] Yan HB, Wang XF, Zhang Q, Tang ZQ, Jiang YH, Fan HZ, Sun YH, Yang PY, Liu F (2014). Reduced expression of the chromatin remodeling gene ARID1A enhances gastric cancer cell migration and invasion via downregulation of E-cadherin transcription. Carcinogenesis.

[32] Qiao J, Cui SJ, Xu LL, Chen SJ, Yao J, Jiang YH, Peng G, Fang CY, Yang PY, Liu F (2015). Filamin C, a dysregulated protein in cancer revealed by label-free quantitative proteomic analyses of human gastric cancer cells. Oncotarget.

