# Supplementary material for: Epithelial but not stromal expression of collagen alpha-1(III) is a diagnostic and prognostic indicator of colorectal carcinoma
Source: Oncotarget. 2016 Jan 3;7(8):8823–38. doi: 10.18632/oncotarget.6815 (PMC4891007; doi:10.18632/oncotarget.6815)
Supplement: Supplementary file 1 [file oncotarget-07-8823-s001.pdf]

Epithelial but not stromal expression of collagen alpha-1(III) is a diagnostic and prognostic indicator of colorectal carcinoma

Supplementary Material

Supplementary Table 1. The clinicopathological factors of the colorectal cancer patients in the tissue microarray.

The tissue microarray is a commercial product of Shanghai Outdo Biotech, China (catalog no. HCol- Ade180Sur-04).

[a]The expression of COL3A1 was represented by the positivity calculated by the positive pixel count algorithm (v9.1) provided by the Aperio ImageScope v12.1.

Code	Operation time	Survival state	Follow-up time	Survival time	Sex	Age	Pathological type	Metastasis	Size	Grade	Volume (cm3)	Vascular Invasion Present	Lymphatic Invation Present	Lymph node number	Positive lymph node	T	N	M	The AJCC Cancer Staging, 7th Edition	Cancer epithelium (Positivity)a	Normal epithelium(Positivity)	Cancer stroma (Positivity)	Normal stroma (Positivity)
RDgCol0609A0277	2006.7	Alive	2014.8	97	M	62	Adenocarcinoma	No	4×3.5×1.5cm	II	21	-	-	5	0	T3	N0	M0	2A	0.877	0.801	0.593	0.467
RDgCol0609A0278	2006.7	Dead	2009/11/19	40	M	75	Adenocarcinoma	No	2.5×2.5×1cm	II-III	6.25	-	-	-	-	T4b	N0	M0	2C	n/a	0.599	n/a	0.566
RDgCol0609A0280	2006.7	Alive	2014.8	97	F	81	Adenocarcinoma	No	9×7×3cm	II	189	-	-	-	-	T3	N0	M0	2A	0.902	0.328	0.829	0.462
RDgCol0609A0281	2006.7	Dead	2009/2/17	31	M	70	Adenocarcinoma	No	6×5.5×1，7×5×0.5cm	II	50.5	-	-	-	-	T3	N0	M0	2A	0.907	0.694	0.649	0.528
RDgCol0609A0283	2006.8	Dead	2006/11/30	3	F	73	Adenocarcinoma	Yes	5×3.5×3cm	II-III	52.5	-	positive	6	3	T3	N1b	M1b	4B	0.907	0.871	0.535	0.640
RDgCol0609A0284	2006.8	Alive	2014.8	96	M	71	Adenocarcinoma	No	6×4×1.5cm	II	36	-	-	-	-	T3	N0	M0	2A	0.866	0.645	0.418	0.532
RDgCol0609A0285	2006.8	Dead	2011/3/23	55	F	70	Adenocarcinoma	No	5.5×4.5×0.7cm	II-III	17.325	-	positive	3	1	T3	N1a	M0	3B	0.925	n/a	0.646	n/a

RDgCol0609A0287	2006.8	Alive	2014.8	96	M	55	Adenocarcinoma	No	7.5×4.5×2.5cm	II-III	84.375	-	-	-	-	T3	N0	M0	2A	0.907	0.724	0.696	0.475
RDgCol0609A0288	2006.8	Dead	2009/2/12	30	F	58	Adenocarcinoma	No	5×4×1.5cm	II-III	30	-	positive	7	4	T3	N2a	M0	3B	0.917	0.598	0.628	0.446
RDgCol0609A0289	2006.8	Dead	2007/1/25	5	M	72	Adenocarcinoma	No	4×2.5×2cm	I-III	20	-	-	-	-	T3	N0	M0	2A	n/a	0.739	n/a	n/a
RDgCol0609A0291	2006.9	Alive	2014.8	95	F	62	Adenocarcinoma	No	7.5×5×2cm	II-III	75	-	-	10	0	T3	N0	M0	2A	0.840	n/a	0.753	n/a
RDgCol0609A0292	2006.9	Alive	2014.8	95	M	85	Adenocarcinoma	No	4×4×2.5cm	II-III	40	-	-	9	0	-	N0	M0	-	0.851	n/a	0.555	0.418
RDgCol0609A0293	2006.9	Dead	2011/11/27	62	F	75	Adenocarcinoma	No	4.5×3×1.5cm	II	20.25	-	positive	2	2	T3	N1b	M0	3B	0.892	0.739	0.618	0.682
RDgCol0609A0294	2006.9	Dead	2008/3/3	18	M	48	Adenocarcinoma	No	5.5×4×2cm	II-III	44	-	positive	9	6	T3	N2a	M0	3B	0.885	0.863	0.594	0.468
RDgCol0609A0295	2006.9	Dead	2010/12/5	51	F	24	Adenocarcinoma	No	3.5×3×1cm	II-III	10.5	-	positive	24	4	T3	N2a	M0	3B	0.905	0.888	0.690	0.657
RDgCol0609A0296	2006.9	Dead	2011/4/12	55	M	80	Adenocarcinoma	No	4×3×1.5，2 × 1.7 × 1cm	II	21.4	-	-	-	-	T3	N0	M0	2A	0.840	0.739	0.638	0.410
RDgCol0609A0297	2006.9	Dead	2008/9/30	24	M	75	Adenocarcinoma	No	7×6×2cm	II	84	-	-	7	2	T4a	N1b	M0	3B	0.880	0.754	0.777	0.598
RDgCol0609A0298	2006.9	Alive	2014.8	95	F	59	Adenocarcinoma	No	4.5×3×1.5cm	II-III	20.25	-	-	4	0	T3	N0	M0	2A	0.927	0.640	0.722	0.628
RDgCol0611A0309	2006.9	Dead	2008/11/3	26	M	83	Adenocarcinoma	No	7.5×5×2cm	II	75	-	positive	6	2	T4b	N1b	M0	3C	0.846	0.739	0.242	0.567
RDgCol0611A0310	2006.9	Dead	2007/2/19	5	F	73	Adenocarcinoma	No	6×5×3cm	III	90	-	positive	8	6	T3	N2a	M0	3B	0.858	0.701	0.582	0.642
RDgCol0611A0311	2006.9	Dead	2008/10/16	25	F	52	Adenocarcinoma	No	5×4×2.5cm	II	50	positive	positive	25	6	T3	N2a	M0	3B	0.906	0.837	0.613	0.605
RDgCol0611A0312	2006.9	Alive	2014.8	95	M	80	Adenocarcinoma	No	5×4.5×1cm	II-III	22.5	-	-	5	0	T3	N0	M0	2A	0.885	0.817	0.484	0.641
RDgCol0701A0404	2007.1	Dead	2007/7/13	6	M	80	Adenocarcinoma	No	9×8×7cm	III	504	-	-	10	0	T4b	N0	M0	2C	0.897	0.816	0.658	0.604
RDgCol0701A0405	2007.1	Dead	2008/1/7	12	F	62	Adenocarcinoma	No	10×10×9cm	III	900	-	-	19	10	T3	N2b	M0	3C	0.816	0.694	0.599	0.481
RDgCol0704A	2007.	Dea	2011/3/	50	M	6	Adenocarcin	No	1-4×3×3cm	II	-	-	-	6	0	T3	N0	M0	2A	0.906	0.510	0.517	0.435

0431	1	d	9			2	oma																
RDgCol0704A0435	2007.1	Dead	2008/5/14	16	M	成	Adenocarcinoma	No	5.5 × 4.5 × 3.5cm	II-III	86.625	-	-	10	0	T3	N0	M0	2A	0.938	0.618	0.687	0.501
RDgCol0704A0436	2007.1	Alive	2014.8	91	F	成	Adenocarcinoma	No	3.5 × 2 × 2cm	II-III	14	-	-	5	0	T3	N0	M0	2A	0.854	0.345	0.645	0.628
RDgCol0704A0437	2007.1	Dead	2008/7/10	18	F	71	Adenocarcinoma	No	7×7×4cm	II-III	196	-	-	10	0	T2	N0	M0	1	0.828	0.649	0.394	0.676
RDgCol0704A0438	2007.1	Alive	2014.8	91	F	70	Adenocarcinoma	No	7×7×4cm	II	196	-	-	6	2	T3	N1b	M0	3B	0.849	0.739	0.610	0.548
RDgCol0704A0449	2007.1	Alive	2012/8/16		M	78	Adenocarcinoma	No	2-1.2×1.5×1.5cm	II		-	-	-		T4a	N0	M0	2B	0.845	0.474	0.551	0.604
RDgCol0704A0439	2007.2	Alive	2014.8	90	M	63	Adenocarcinoma	No	9×8×8cm	II	576	-	-	12	0	T3	N0	M0	2A	0.798	0.798	0.517	0.561
RDgCol0704A0440	2007.2	Dead	2008/10/6	20	M	74	Adenocarcinoma	No	15×13×11cm	II	2145	-	-	6	1	T2	N1a	M0	3A	0.948	0.668	0.669	0.563
RDgCol0704A0441	2007.2	Dead	2008/6/26	16	F	79	Adenocarcinoma	No	6×6×5cm	II	180	-	-	5	2	T4b	N1b	M0	3C	0.921	0.859	0.574	0.687
RDgCol0704A0442	2007.2	Dead	2012/12/17	70	M	89	Adenocarcinoma	No	15×13×11cm	II-III	2145	-	-	6	0	T3	N0	M0	2A	0.877	n/a	0.396	n/a
RDgCol0704A0444	2007.2	Dead	2008/11/5	21	F	62	Adenocarcinoma	No	4 × 3 × 2.5cm， 2.5×2×1.5cm	II	37.5	positive	positive	16	12	T3	N2b	M0	3C	0.927	0.783	0.538	0.599
RDgCol0704A0445	2007.2	Alive	2014.8	90	F	62	Adenocarcinoma	No	15 × 10 × 10cm，2×2×1.5cm	II	1506	-	-	10	0	-	N0	-	-	0.861	0.793	0.614	0.622
RDgCol0704A0447	2007.2	Dead	2011/6/26	52	F	80	Adenocarcinoma	No	4×4×1.5cm	II	24	-	-	8	0	T3	N0	M0	2A	0.895	0.495	0.185	0.344
RDgCol0704A0475	2007.2	Dead	2010/2/14	36	M	90	Adenocarcinoma	No	5×4×1cm	II-III	20	-	-	6	0	T3	N0	M0	2A	0.873	n/a	0.638	0.682
RDgCol0704A0476	2007.2	Dead	2007/7/15	5	M	48	Adenocarcinoma	No	8×7×3cm	III	168	-	-	5	0	T4b	N0	M0	2C	0.823	0.830	0.755	0.552
RDgCol0704A0451	2007.3	Alive	2012/8/16		F	68	Adenocarcinoma	No	3.5×3×2cm	III	21	-	-	39	0	T3	N0	M0	2A	0.906	0.735	0.541	0.681
RDgCol0704A0452	2007.3	Dead	2008/1/22	10	F	67	Adenocarcinoma	No	15 × 10 × 10cm，2×2×1.5cm	II	1506	-	-	4	1	T4a	N1a	M0	3B	0.907	0.715	0.645	0.569

RDgCol0704A0453	2007.3	Dead	2008/5/29	14	F	84	Adenocarcinoma	No	6×5×5cm	II-III	150	-	-	5	1	T3	N1a	M0	3B	0.918	0.702	0.718	0.678
RDgCol0704A0454	2007.3	Alive	2012/8/16		M	73	Adenocarcinoma	No	4.5×3.5×1.5cm	II-III	23.625	-	-	7	0	T3	N0	M0	2A	0.923	0.648	0.399	0.554
RDgCol0704A0455	2007.3	Dead	2011/4/9	49	F	78	Adenocarcinoma	No	3.5×3×1cm	II	10.5	-	-	5	1	T3	N1a	M0	3B	0.924	0.635	0.465	0.557
RDgCol0704A0457	2007.3	Alive	2012/8/16		M	65	Adenocarcinoma	No	4.5×4×1cm	II	18	-	-	8	0	T3	N0	M0	2A	0.927	0.452	0.636	0.449
RDgCol0704A0459	2007.3	Alive	2014.8	89	M	65	Adenocarcinoma	No	5.5×3×0.8cm	II	13.2	-	-	3	0	T3	N0	M0	2A	0.901	0.692	0.700	0.535
RDgCol0704A0460	2007.3	Dead	2012/5/23	62	M	80	Adenocarcinoma	No	8×5×5cm	II	200	-	-	8	0	T3	N0	M0	2A	0.795	0.740	0.494	0.602
RDgCol0704A0461	2007.3	Alive	2014.8	89	F	79	Adenocarcinoma	No	3×3×2cm	II	18	-	-	14	2	T3	N1b	M0	3B	0.921	0.791	0.365	0.599
RDgCol0704A0463	2007.3	Dead	2010/11/2	44	M	67	Adenocarcinoma	No	3.5×3×2cm	II-III	21	-	-	8	3	T3	N1b	M0	3B	0.887	0.738	0.655	0.573
RDgCol0704A0464	2007.3	Alive	2012/8/16		M	67	Adenocarcinoma	No	15×13×11cm	II	2145	-	-	10	0	T3	N0	M0	2A	0.825	0.604	0.367	0.514
RDgCol0704A0465	2007.3	Dead	2010/4/22	37	M	72	Adenocarcinoma	No	10×10×8.2cm	II	820	-	-	14	1	T3	N1a	M0	3B	n/a	0.672	n/a	0.571
RDgCol0704A0466	2007.3	Alive	2014.8	89	M	62	Adenocarcinoma	No	6×6×3cm	II	108	-	-	9	0	T3	N0	M0	2A	0.920	0.705	0.695	0.585
RDgCol0705A0524	2007.3	Alive	2014/2/21	83	F	82	Adenocarcinoma	No	9×5.5×3cm	I	148.5	negative	-	2	0	T2	N0	M0	1	0.753	0.591	0.455	0.566
RDgCol0705A0525	2007.4	Alive	2014.8	88	F	57	Adenocarcinoma	No	4.5×3×1cm	II-III	13.5	negative	positive	12	1	T3	N1a	M0	3B	0.891	0.606	0.220	0.639
RDgCol0705A0526	2007.4	Dead	2008/9/19	17	M	73	Adenocarcinoma	Yes	5.5×4.5×2.5cm	I	61.875	positive	-	7	1	T1	N1a	M1a	4A	0.886	0.592	0.592	0.370
RDgCol0705A0528	2007.4	Alive	2014.8	88	M	78	Adenocarcinoma	No	2.7×2.5×2cm	I-III	13.5	negative	-	4	0	T1	N0	M0	1	0.857	0.794	0.500	0.637
RDgCol0705A0529	2007.4	Alive	2014.8	88	F	77	Adenocarcinoma	No	-	II	-	negative	-	4	0	T3	N0	M0	2A	n/a	0.707	0.717	0.548
RDgCol0705A0530	2007.4	Alive	2014.8	88	F	84	Adenocarcinoma	No	2×2×1.5cm	II	6	negative	-	5	0	T2	N0	M0	1	0.874	0.786	0.519	0.655
RDgCol0705A0531	2007.4	Alive	2014.8	88	M	80	Adenocarcinoma	No	10×6×1cm	III	60	negative	-	9	2	T3	N1b	M0	3B	0.943	0.651	0.642	0.355

RDgCol0705A0532	2007.4	Alive	2014.8	88	F	65	Adenocarcinoma	No	5.5×4×1cm	II	22	negative	-	11	0	T3	N0	M0	2A	0.936	n/a	0.707	n/a
RDgCol0705A0533	2007.4	Dead	2010/2/5	34	F	76	Adenocarcinoma	No	8×4.5×2cm	II	72	negative	-	9	0	T3	N0	M0	2A	0.906	0.551	0.588	0.570
RDgCol0705A0534	2007.4	Alive	2012/8/16		F	60	Adenocarcinoma	No	4×2×1.3cm	II	10.4	negative	-	4	2	T3	N1b	M0	3B	0.826	0.592	0.549	0.451
RDgCol0705A0535	2007.4	Alive	2014/2/9	82	M	74	Adenocarcinoma	No	5×4×1.5cm	II	30	negative	-	6	0	T3	N0	M0	2A	0.917	0.540	0.859	0.513
RDgCol0705A0536	2007.4	Alive	2012/8/16		M	67	Adenocarcinoma	No	12×11×5cm	II-III	660	negative	-	11	0	T4b	N0	M0	2A	0.953	0.605	0.727	0.511
RDgCol0705A0538	2007.4	Dead	2011/12/1	56	M	74	Adenocarcinoma	No	4 × 3 × 1.5cm， 2.8×2×2cm	I-II	29.2	negative	-	13	0	T3	N0	M0	2A	0.920	0.766	0.550	0.400
RDgCol0705A0539	2007.4	Dead	2008/7/25	15	F	71	Adenocarcinoma	No	5 × 4 × 1.3cm，5×3.5×1cm	II-III	43.5	negative	positive	6	4	T3	N2a	M0	3B	0.961	0.598	0.575	0.604
RDgCol0705A0540	2007.4	Alive	2014.8	88	F	54	Adenocarcinoma	No	4×3.5×1cm	II-III	14	negative	positive	10	1	T4a	N1a	M0	3B	0.953	n/a	0.510	0.592
RDgCol0705A0541	2007.4	Dead	2009/4/19	24	F	67	Adenocarcinoma	No	4×3×1.5cm	II-III	18	negative	-	7	2	T3	N1b	M0	3B	0.966	0.746	0.801	0.639
RDgCol0705A0543	2007.4	Dead	2010/2/25	34	F	63	Adenocarcinoma	No	3.5×3×1.5cm	II	15.75	negative	-	12	1	T3	N1a	M0	3B	n/a	0.633	n/a	0.621
RDgCol0705A0544	2007.4	Alive	2014.8	88	M	58	Adenocarcinoma	No	5×5×1.5cm	I-II	37.5	negative	-	3	0	T3	N0	M0	2A	0.887	n/a	0.558	0.619
RDgCol0706A0575	2007.4	Alive	2013/12/14	80	M	62	Adenocarcinoma	No	2.3×2×0.5cm	II	2.3	negative	-	4	0	T2	N0	M0	1	0.848	0.814	0.838	0.613
RDgCol0706A0576	2007.5	Alive	2014.8	87	F	54	Adenocarcinoma	No	4×4×1cm	II	16	negative	-	7	0	T3	N0	M0	2A	0.914	0.801	0.794	0.533
RDgCol0706A0577	2007.5	Dead	2008/4/29	11	M	76	Adenocarcinoma	No	3.5 × 3 × 1cm，7×6×3cm	II	136.5	negative	-	4	0	T3	N0	M0	2A	0.934	0.663	0.720	0.474
RDgCol0706A0579	2007.5	Dead	2007/11/29	6	F	73	Adenocarcinoma	No	5×4×1.3cm	II	26	negative	-	-	0	T3	N1c	M0	3B	0.968	0.761	0.314	0.643
RDgCol0706A0583	2007.5	Dead	2009/9/3	28	M	64	Adenocarcinoma	No	5.5×4×2.5cm	II-III	55	negative	-	12	0	T3	N0	M0	2A	0.877	0.778	0.645	0.718
RDgCol0706A	2007.	Alive	2014.8	87	M	5	Adenocarcin	No	6×5×1.5cm	I-II	45	negati	-	5	0	T3	N0	M0	2A	0.966	0.462	0.584	n/a

0584	5					5	oma					ve											
RDgCol0706A0585	2007.5	Alive	2014.8	87	M	47	Adenocarcinoma	No	3×2×1.5cm	I	9	negative	-	8	0	T1	N0	M0	1	0.875	0.819	0.749	0.558
RDgCol0706A0586	2007.5	Alive	2014.8	87	F	63	Adenocarcinoma	No	4.5×4×1cm	II	18	negative	-	8	2	T3	N1b	M0	3B	0.905	0.658	0.337	0.640
RDgCol0706A0587	2007.5	Dead	2012/10/17	65	M	87	Adenocarcinoma	No	4.5×4×2cm	I	36	negative	-	2	0	T3	N0	M0	2A	0.823	0.602	0.413	n/a
RDgCol0706A0588	2007.5	Dead	2010/6/24	37	M	66	Adenocarcinoma	No	2.5×2×1cm	II	5	negative	-	10	0	T4a	N0	M0	2B	0.896	0.710	0.720	0.478
RDgCol0706A0589	2007.5	Dead	2008/8/6	15	M	63	Adenocarcinoma	No	7.5×5.5×2cm	II	82.5	negative	-	8	0	T3	N0	M0	2A	0.911	0.699	0.305	0.534
RDgCol0706A0590	2007.5	Alive	2014.8	87	F	58	Adenocarcinoma	No	3.5×2×1.5cm	II	10.5	negative	-	-	-	T3	N0	M0	2A	0.912	0.759	0.419	0.517
RDgCol0706A0591	2007.5	Alive	2014.8	87	F	75	Adenocarcinoma	No	7×4×1cm	II	28	negative	-	5	0	T3	N0	M0	2A	0.892	0.737	0.585	0.657
RDgCol0706A0592	2007.5	Alive	2014.8	87	F	70	Adenocarcinoma	No	2.2×2×1cm	II	4.4	negative	-	8	0	T3	N0	M0	2A	0.858	0.830	0.714	0.512
RDgCol0706A0593	2007.5	Dead	2010/3/29	34	M	84	Adenocarcinoma	No	9×8×3cm	II	216	negative	-	1	0	T3	N0	M0	2A	n/a	0.592	n/a	0.549
RDgCol0706A0596	2007.5	Alive	2014.8	87	F	64	Adenocarcinoma	No	5×5×1.5cm	I	37.5	negative	-	2	1	T3	N1a	M0	3B	0.890	0.582	0.655	0.560
RDgCol0706A0597	2007.5	Alive	2014.8	87	F	58	Adenocarcinoma	No	3.5×3×1cm	II-III	10.5	negative	-	4	2	T3	N1b	M0	3B	0.881	0.770	0.422	0.716
RDgCol0706A0606	2007.5	Alive	2014.8	87	F	74	Adenocarcinoma	No	6.5×6×4.5cm	II	175.5	negative	-	11	0	T2	N0	M0	1	0.855	0.651	0.465	0.602
RDgCol0706A0607	2007.5	Alive	2014.8	87	M	66	Adenocarcinoma	No	-	II-III	-	negative	-	20	0	T3	N0	M0	2A	0.877	0.622	0.551	0.631
RDgCol0706A0609	2007.5	Dead	2008/7/29	14	M	62	Adenocarcinoma	No	6×5×1.3cm	II-III	39	negative	-	7	4	T3	N2a	M0	3B	0.863	0.650	0.654	0.552

Supplementary Table 2. Correlation of COL3A1 protein expression in cancer stromal cells with clinicopathological variables in CRC patients revealed by TMA-IHC analysis.

[a]The epithelial cell-specific expression of COL3A1 was dichotomized into low (L) or high (H) groups based on the positivity values calculated by the positive pixel count algorithm (v9.1) provided by the Aperio ImageScope v12.1.

**Clinicopathological parameters**	**Total cases**	**COL3A1 protein[a]**		**χ2**	***p* value**
		**Low**	**High**		
Gender					
Male	43	22	21	0.11	0.74
Female	42	23	19		
Age					
< 70 years	41	19	22	2.025	0.155
≥70 years	42	26	16		
Grade					
I	5	3	2	0.883	0.682
I-II, II	46	26	20		
I-III, II-III, III	34	16	18		
Vascular Invasion Present					
positive	3	2	1	0.103	1
negative	35	20	15		
Number of Positive Lymph Nodes					
< 1	47	26	21	0.103	0.748
≥ 1	31	16	15		
T stage					
T1-T2	9	6	3	1.338	0.558
T3	64	34	30		
T4	10	4	6		
N stage					
N0	53	28	25	0.001	0.979
N1-N2	32	17	15		
M stage					
M0	82	43	39	1.776	0.497
M1	2	2	0		
Stage					
1-2	51	27	24	1.531	0.572
3	15	15	15		
4	2	2	0		

Metastasis					
No	83	43	40	1.821	0.496
Yes	2	2	0		
Tumor volume (cm3)					
< 70	56	32	24	1.179	0.278
≥ 70	27	12	15		

Supplementary Table 3. Plasma samples used for ELISA assay of COL3A1 and CEA.

No.	Sample types	Sex	Age	Smoking history	Family history	Clinical diagnosis	Pathologic diagnosis	Tumor size	Tumor stage	Degree of differentiation	Lymph node metastasis	Serum concentration of COL3A1(ng/ml)	Serum concentration of CEA(ng/ml)
1	Colon cancer	Female	53	No	No	Colon cancer	Adenocarcinoma	5.5*5*1.3	T3N2M1	Moderately differentiation	Yes	60.72	n/a
2	Colon cancer	Male	59	No	No	Colon cancer	Adenocarcinoma	7*3.6*0.7	T3N2M0	Moderately differentiation	Yes	69.16	n/a
3	Colon cancer	Male	38	No	No	Colon cancer	n/a	5*4*3	T3N2M0	n/a	Yes	69.97	n/a
4	Colon cancer	Male	56	Yes	No	Colon cancer	Adenocarcinoma	5.5*5*0.6	T3N0M0	Moderately differentiation	No	87.05	n/a
5	Colon cancer	Female	61	No	No	Colon cancer	Adenocarcinoma	5*5*2.5	T3N0M0	Moderately differentiation	No	84.60	n/a
6	Colon cancer	Male	65	No	No	Colon cancer	Adenocarcinoma	5*5.4*0.5	T3N0M0	Moderately differentiation	No	61.92	n/a
7	Colon cancer	Male	48	No	No	Colon cancer	Adenocarcinoma	4*2.5*1.5	T2N0M0	Moderately differentiation	No	73.61	n/a
8	Colon cancer	Male	52	Yes	No	Colon cancer	n/a	6*3.5*1.1	T3N2M0	Moderately differentiation	Yes	64.73	n/a
9	Colon cancer	Male	82	No	No	Colon cancer	Adenocarcinoma	4*2*1.5	T3N0M0	Moderately differentiation	No	77.67	n/a
10	Colon cancer	Male	51	No	No	Colon cancer	Adenocarcinoma	4*4*1.2	T3N1M0	Moderately differentiation	Yes	57.12	n/a
11	Colon cancer	Male	52	Yes	No	Colon cancer	Adenocarcinoma	5*4.5*1.5	T2N0M0	Moderately differentiation	No	61.52	n/a
12	Colon cancer	Male	72	No	No	Colon cancer	Adenocarcinoma	3.5*3*12*1.5*0.9	T4N1M0	Moderately differentiation	Yes	73.61	n/a
13	Colon cancer	Female	39	No	No	Colon cancer	Adenocarcinoma	3.4*3.2*2	T4N2M0	Moderately differentiation	Yes	58.72	n/a
14	Colon cancer	Male	52	Yes	No	Colon cancer	Adenocarcinoma	7.5*5.4*1.8	T4N0M0	Moderately differentiation	No	62.72	n/a
15	Colon cancer	Female	43	No	No	Colon cancer	n/a	n/a	T3N0M0	Moderately differentiation	No	64.33	n/a
16	Colon cancer	Male	70	No	No	Colon cancer	Adenocarcinoma	3*3*0.5	T3N2M0	Low differentiation	Yes	54.72	n/a

17	Colon cancer	Fem ale	61	No	No	Colon cancer	Adenocarcino ma	3.5*3.5* 1.2	T4N1M0	Moderately differentiation	Yes	66.75	n/a
18	Colon cancer	Mal e	68	No	No	Colon cancer	n/a	6.2*5*2	T3N0M0	Moderately differentiation	No	61.92	n/a
19	Colon cancer	Mal e	57	Yes	No	Colon cancer	Adenocarcino ma	3*2.5*2. 2	T4N0M0	Moderately differentiation	No	56.32	n/a
20	Colon cancer	Fem ale	73	No	No	Colon cancer	Adenocarcino ma	3*3*0.8	T4N2M0	Low differentiation	Yes	57.92	n/a
21	Colon cancer	Fem ale	39	No	No	Colon cancer	Adenocarcino ma	3.4*3.2* 2	T4N2M0	Moderately differentiation	Yes	78.89	n/a
22	Colon cancer	Mal e	61	No	No	Colon cancer	Adenocarcino ma	3*3.3*0. 7	T3M0N0	Moderately differentiation	No	70.78	n/a
23	Colon cancer	Fem ale	69	No	No	Colon cancer	Adenocarcino ma	5*4.5*0. 6	T2N1M0	Moderately differentiation	Yes	53.13	n/a
24	Colon cancer	Mal e	73	No	No	Colon cancer	Adenocarcino ma	2.4*2*0. 8	T2N2M0	Low differentiation	Yes	65.94	n/a
25	Colon cancer	Fem ale	67	No	No	Colon cancer	n/a	n/a	T2N4M0	Low differentiation	Yes	58.32	n/a
26	Colon cancer	Mal e	73	No	Yes	Colon cancer	Adenocarcino ma	4*4*1	T3N0M0	High differentiation	No	65.14	n/a
27	Colon cancer	Mal e	65	No	No	Colon cancer	Adenocarcino ma	5*4*2	T3N0M0	Moderately differentiation	No	71.99	n/a
28	Colon cancer	Fem ale	53	No	No	Colon cancer	Adenocarcino ma	2.5*2*0. 5	T4N0M0	Moderately differentiation	No	63.53	n/a
29	Colon cancer	Mal e	69	No	No	Colon cancer	Adenocarcino ma	2.5*2*0. 5	T2N0M0	Moderately differentiation	No	70.38	n/a
30	Colon cancer	Mal e	67	No	No	Colon cancer	Adenocarcino ma	3*2.3*1	T3N1M0	Moderately differentiation	Yes	55.92	n/a
31	Colon cancer	Mal e	87	n/a	n/a	Colon cancer	Adenocarcino ma	7.5*5.5* 2.5	n/a	Moderately differentiation	No	198.82	n/a
32	Colon cancer	Fem ale	70	n/a	n/a	Colon cancer	Adenocarcino ma	5*4.5*0. 5	n/a	Moderately differentiation	Yes	97.33	n/a
33	Colon cancer	Mal e	60	n/a	n/a	Colon cancer	Adenocarcino ma	5*4*3	n/a	Moderately differentiation	No	152.51	n/a
34	Colon cancer	Mal e	79	n/a	n/a	Colon cancer	n/a	7*5*0.8	n/a	Low differentiation	No	123.59	1.00
35	Colon cancer	Mal e	71	n/a	n/a	Colon cancer	n/a	4.5*3*3	n/a	Moderately differentiation	No	110.18	1.96
36	Colon	Fem	54	n/a	n/a	Colon	n/a	1.6*1.6*	n/a	Low	Yes	102.70	0.24

	cancer	ale				cancer		0.5		differentiation			
37	Colon cancer	Male	74	n/a	n/a	Colon cancer	n/a	4*3*1.5	n/a	Moderately differentiation	Yes	98.57	n/a
38	Colon cancer	Male	42	n/a	n/a	Colon cancer	n/a	n/a	n/a	n/a	n/a	113.52	n/a
39	Colon cancer	Male	63	n/a	n/a	Colon cancer	n/a	6*6	n/a	Moderately differentiation	Yes	124.85	113.44
40	Colon cancer	Male	59	n/a	n/a	Colon cancer	n/a	n/a	n/a	n/a	n/a	186.00	119.28
41	Colon cancer	n/a	n/a	n/a	n/a	Colon cancer	n/a	n/a	n/a	n/a	n/a	n/a	6.28
42	Colon cancer	n/a	n/a	n/a	n/a	Colon cancer	n/a	n/a	n/a	n/a	n/a	n/a	1.70
43	Colon cancer	n/a	n/a	n/a	n/a	Colon cancer	n/a	n/a	n/a	n/a	n/a	n/a	93.19
44	Colon cancer	n/a	n/a	n/a	n/a	Colon cancer	n/a	n/a	n/a	n/a	n/a	n/a	1.25
45	Colon cancer	n/a	n/a	n/a	n/a	Colon cancer	n/a	n/a	n/a	n/a	n/a	n/a	8.37
46	Colon cancer	n/a	n/a	n/a	n/a	Colon cancer	n/a	n/a	n/a	n/a	n/a	n/a	7.13
47	Colon cancer	Female	73	No	No	Colon cancer	Adenocarcinoma	n/a	n/a	Moderately differentiation	No	71.99	1.20
48	Colon cancer	Female	83	No	No	Colon cancer	n/a	n/a	n/a	n/a	Yes	54.72	1.91
49	Colon cancer	Male	77	No	No	Colon cancer	n/a	n/a	n/a	n/a	n/a	n/a	3.15
50	Colon cancer	Male	72	No	No	Colon cancer	Adenocarcinoma	n/a	n/a	n/a	n/a	142.66	3.12
51	Colon cancer	Female	80	No	No	Colon cancer	n/a	n/a	n/a	n/a	Yes	104.78	42.76
52	Colon cancer	Male	78	n/a	n/a	Colon cancer	Adenocarcinoma	n/a	n/a	n/a	Yes	166.13	1.96
53	Colon cancer	Female	61	n/a	n/a	Colon cancer	Adenocarcinoma	n/a	n/a	n/a	Yes	478.13	94.14
54	Colon cancer	Male	64	n/a	n/a	Colon cancer	Adenocarcinoma	n/a	n/a	n/a	Yes	129.58	5.27
55	Colon cancer	Female	57	n/a	n/a	Colon cancer	Adenocarcinoma	n/a	n/a	n/a	No	155.68	6.63

56	Colon cancer	Male	47	n/a	n/a	Colon cancer	Adenocarcinoma	n/a	n/a	n/a	Yes	173.70	1.45
57	Colon cancer	Male	82	n/a	n/a	Colon cancer	Adenocarcinoma	n/a	n/a	n/a	Yes	216.08	100.90
58	Colon cancer	Male	82	n/a	n/a	Colon cancer	Adenocarcinoma	n/a	n/a	n/a	Yes	494.24	2.05
59	Colon cancer	Male	55	n/a	n/a	Colon cancer	Adenocarcinoma	n/a	n/a	n/a	No	129.29	1.80
60	Colon cancer	Male	60	n/a	n/a	Colon cancer	Adenocarcinoma	n/a	n/a	n/a	No	228.80	2.78
61	Colon cancer	Female	55	n/a	n/a	Colon cancer	Adenocarcinoma	n/a	n/a	n/a	No	189.41	1.05
62	Colon cancer	Male	71	n/a	n/a	Colon cancer	Adenocarcinoma	n/a	n/a	n/a	No	355.77	0.69
63	Colon cancer	Female	40	n/a	n/a	Colon cancer	Adenocarcinoma	n/a	n/a	n/a	Yes	209.97	0.82
64	Colon cancer	Female	63	n/a	n/a	Colon cancer	Adenocarcinoma	n/a	n/a	n/a	Yes	221.93	3.66
65	Colon cancer	Male	66	n/a	n/a	Colon cancer	Adenocarcinoma	n/a	n/a	n/a	No	278.63	2.31
66	Colon cancer	Female	69	n/a	n/a	Colon cancer	Adenocarcinoma	n/a	n/a	n/a	No	650.57	1.00
67	Colon cancer	Female	73	n/a	n/a	Colon cancer	Adenocarcinoma	n/a	n/a	n/a	No	236.73	0.76
68	Colon cancer	Female	77	n/a	n/a	Colon cancer	Adenocarcinoma	n/a	n/a	n/a	Yes	143.94	72.61
69	Colon cancer	Male	73	n/a	n/a	Colon cancer	Adenocarcinoma	n/a	n/a	n/a	No	145.10	0.71
70	Colon cancer	Female	70	n/a	n/a	Colon cancer	Adenocarcinoma	n/a	n/a	n/a	Yes	270.29	0.54
71	Colon cancer	Male	65	n/a	n/a	Colon cancer	Adenocarcinoma	n/a	n/a	n/a	No	155.08	0.87
72	Colon cancer	Female	76	n/a	n/a	Colon cancer	Adenocarcinoma	n/a	n/a	n/a	n/a	180.74	12.63
73	Colon cancer	Male	71	n/a	n/a	Colon cancer	Adenocarcinoma	n/a	n/a	n/a	No	250.80	54.96
74	Colon cancer	Male	72	n/a	n/a	Colon cancer	Adenocarcinoma	n/a	n/a	n/a	No	111.70	0.34
75	Colon	Fem	81	n/a	n/a	Colon	Adenocarcino	n/a	n/a	n/a	No	164.33	24.72

	cancer	ale				cancer	ma						
76	Colon cancer	Male	52	n/a	n/a	Colon cancer	Adenocarcinoma	n/a	n/a	n/a	Yes	215.44	14.32
77	Colon cancer	Male	59	n/a	n/a	Colon cancer	Adenocarcinoma	n/a	n/a	n/a	No	107.87	n/a
78	Colon cancer	Male	59	n/a	n/a	Colon cancer	Adenocarcinoma	n/a	n/a	n/a	Yes	136.14	12.54
79	Colon cancer	Male	57	n/a	n/a	Colon cancer	Adenocarcinoma	n/a	n/a	n/a	No	76.64	4.29
80	Colon cancer	Male	43	n/a	n/a	Colon cancer	Adenocarcinoma	n/a	n/a	n/a	No	223.88	2.12
81	Colon cancer	Male	61	n/a	n/a	Colon cancer	Adenocarcinoma	n/a	n/a	n/a	n/a	245.41	5.29
82	Colon cancer	Male	66	n/a	n/a	Colon cancer	Adenocarcinoma	n/a	n/a	n/a	Yes	450.23	7.24
83	Colon cancer	Female	42	n/a	n/a	Colon cancer	Adenocarcinoma	n/a	n/a	n/a	No	220.95	0.50
84	Colon cancer	Male	57	n/a	n/a	Colon cancer	Adenocarcinoma	n/a	n/a	n/a	Yes	136.71	18.50
85	Colon cancer	Male	67	n/a	n/a	Colon cancer	Adenocarcinoma	n/a	n/a	n/a	Yes	187.55	23.44
86	Colon cancer	Male	80	n/a	n/a	Colon cancer	Adenocarcinoma	n/a	n/a	n/a	No	123.37	9.44
87	Colon cancer	Male	85	n/a	n/a	Colon cancer	Adenocarcinoma	n/a	n/a	n/a	No	854.19	34.31
88	Colon cancer	Male	70	n/a	n/a	Colon cancer	Adenocarcinoma	n/a	n/a	n/a	No	115.01	1.02
89	Colon cancer	Male	66	n/a	n/a	Colon cancer	Adenocarcinoma	n/a	n/a	n/a	No	159.24	7.69
90	Colon cancer	Female	70	n/a	n/a	Colon cancer	Adenocarcinoma	n/a	n/a	n/a	Yes	303.41	1.29
91	Colon cancer	Male	60	n/a	n/a	Colon cancer	Adenocarcinoma	n/a	n/a	n/a	No	245.07	26.11
92	Colon cancer	Male	64	n/a	n/a	Colon cancer	Adenocarcinoma	n/a	n/a	n/a	No	352.75	2.33
93	Colon cancer	Female	61	n/a	n/a	Colon cancer	Adenocarcinoma	n/a	n/a	n/a	n/a	135.28	2.29
1	Enteritis	Male	64	n/a	n/a	Enteritis	n/a	n/a	n/a	n/a	n/a	13.23	1.16

2	Enteritis	Male	53	n/a	n/a	Enteritis	n/a	n/a	n/a	n/a	n/a	234.38	1.05
3	Enteritis	Female	73	n/a	n/a	Enteritis	n/a	n/a	n/a	n/a	n/a	118.45	0.89
4	Enteritis	Female	42	n/a	n/a	Enteritis	n/a	n/a	n/a	n/a	n/a	14.52	0.39
5	Enteritis	Male	30	n/a	n/a	Enteritis	n/a	n/a	n/a	n/a	n/a	8.34	0.63
6	Enteritis	Male	68	n/a	n/a	Enteritis	n/a	n/a	n/a	n/a	n/a	18.52	0.28
7	Enteritis	Female	53	n/a	n/a	Enteritis	n/a	n/a	n/a	n/a	n/a	14.50	n/a
8	Enteritis	Male	49	n/a	n/a	Enteritis	n/a	n/a	n/a	n/a	n/a	9.98	0.43
9	Enteritis	Female	62	n/a	n/a	Enteritis	n/a	n/a	n/a	n/a	n/a	9.83	3.84
10	Enteritis	Male	50	n/a	n/a	Enteritis	n/a	n/a	n/a	n/a	n/a	8.68	1.23
11	Enteritis	Female	35	n/a	n/a	Enteritis	n/a	n/a	n/a	n/a	n/a	14.55	0.34
12	Enteritis	Female	35	n/a	n/a	Enteritis	n/a	n/a	n/a	n/a	n/a	9.81	0.58
13	Enteritis	Female	48	n/a	n/a	Enteritis	n/a	n/a	n/a	n/a	n/a	15.11	0.58
14	Enteritis	Male	31	n/a	n/a	Enteritis	n/a	n/a	n/a	n/a	n/a	11.30	0.19
15	Enteritis	Female	n/a	n/a	n/a	Crohn'sdisease	n/a	n/a	n/a	n/a	n/a	10.72	2.40
16	Enteritis	Female	n/a	n/a	n/a	Crohn'sdisease	n/a	n/a	n/a	n/a	n/a	8.16	n/a
17	Enteritis	Female	n/a	n/a	n/a	Crohn'sdisease	n/a	n/a	n/a	n/a	n/a	10.29	n/a
18	Enteritis	Female	n/a	n/a	n/a	Crohn'sdisease	n/a	n/a	n/a	n/a	n/a	148.61	0.94
19	Enteritis	Male	n/a	n/a	n/a	Crohn'sdisease	n/a	n/a	n/a	n/a	n/a	10.22	n/a
20	Enteritis	Female	n/a	n/a	n/a	Crohn'sdisease	n/a	n/a	n/a	n/a	n/a	67.25	0.26
21	Enteritis	Mal	n/a	n/a	n/a	Crohn'sdis	n/a	n/a	n/a	n/a	n/a	10.32	n/a

		e				ease							
1	Polyps	n/a	n/a	n/a	n/a	Polyps	n/a	n/a	n/a	n/a	n/a	9.21	2.57
2	Polyps	n/a	n/a	n/a	n/a	Polyps	n/a	n/a	n/a	n/a	n/a	11.12	0.91
3	Polyps	n/a	n/a	n/a	n/a	Polyps	n/a	n/a	n/a	n/a	n/a	23.41	n/a
1	Normal	Male	71	n/a	n/a	n/a	n/a	n/a	n/a	n/a	n/a	46.59	7.21
2	Normal	Male	45	n/a	n/a	n/a	n/a	n/a	n/a	n/a	n/a	72.63	1.50
3	Normal	Male	47	n/a	n/a	n/a	n/a	n/a	n/a	n/a	n/a	34.57	2.64
4	Normal	Female	63	n/a	n/a	n/a	n/a	n/a	n/a	n/a	n/a	84.89	6.77
5	Normal	Male	38	n/a	n/a	n/a	n/a	n/a	n/a	n/a	n/a	77.07	3.00
6	Normal	Female	29	n/a	n/a	n/a	n/a	n/a	n/a	n/a	n/a	35.80	0.24
7	Normal	Male	33	n/a	n/a	n/a	n/a	n/a	n/a	n/a	n/a	27.51	2.21
8	Normal	Female	21	n/a	n/a	n/a	n/a	n/a	n/a	n/a	n/a	26.15	0.19
9	Normal	Female	61	n/a	n/a	n/a	n/a	n/a	n/a	n/a	n/a	62.39	1.02
10	Normal	Female	66	n/a	n/a	n/a	n/a	n/a	n/a	n/a	n/a	95.45	0.52
11	Normal	Female	74	n/a	n/a	n/a	n/a	n/a	n/a	n/a	n/a	30.16	n/a
12	Normal	Male	46	n/a	n/a	n/a	n/a	n/a	n/a	n/a	n/a	46.68	n/a
13	Normal	Female	28	n/a	n/a	n/a	n/a	n/a	n/a	n/a	n/a	61.73	0.21
14	Normal	Male	41	n/a	n/a	n/a	n/a	n/a	n/a	n/a	n/a	63.16	1.66
15	Normal	Female	62	n/a	n/a	n/a	n/a	n/a	n/a	n/a	n/a	41.72	1.18
16	Normal	Female	65	n/a	n/a	n/a	n/a	n/a	n/a	n/a	n/a	31.28	0.43
17	Normal	Female	47	n/a	n/a	n/a	n/a	n/a	n/a	n/a	n/a	53.65	2.76
18	Normal	Fem	49	n/a	n/a	n/a	n/a	n/a	n/a	n/a	n/a	57.66	3.81

		ale											
19	Normal	Male	27	n/a	n/a	n/a	n/a	n/a	n/a	n/a	n/a	74.99	n/a
20	Normal	Female	65	n/a	n/a	n/a	n/a	n/a	n/a	n/a	n/a	23.21	0.17
21	Normal	Female	51	n/a	n/a	n/a	n/a	n/a	n/a	n/a	n/a	40.12	1.50
22	Normal	Female	40	n/a	n/a	n/a	n/a	n/a	n/a	n/a	n/a	56.63	0.85
23	Normal	Male	41	n/a	n/a	n/a	n/a	n/a	n/a	n/a	n/a	43.60	9.68
24	Normal	Male	30	n/a	n/a	n/a	n/a	n/a	n/a	n/a	n/a	35.01	0.58
25	Normal	Female	62	n/a	n/a	n/a	n/a	n/a	n/a	n/a	n/a	47.22	0.58
26	Normal	Male	57	n/a	n/a	n/a	n/a	n/a	n/a	n/a	n/a	29.73	0.94
27	Normal	Female	53	n/a	n/a	n/a	n/a	n/a	n/a	n/a	n/a	53.74	3.00
28	Normal	Female	61	n/a	n/a	n/a	n/a	n/a	n/a	n/a	n/a	73.62	0.56
29	Normal	Male	52	n/a	n/a	n/a	n/a	n/a	n/a	n/a	n/a	47.31	1.32
30	Normal	Male	52	n/a	n/a	n/a	n/a	n/a	n/a	n/a	n/a	55.33	n/a
31	Normal	Male	50	n/a	n/a	n/a	n/a	n/a	n/a	n/a	n/a	37.46	0.60
32	Normal	Male	47	n/a	n/a	n/a	n/a	n/a	n/a	n/a	n/a	42.62	1.52
33	Normal	Female	52	n/a	n/a	n/a	n/a	n/a	n/a	n/a	n/a	28.70	0.37
34	Normal	Female	36	n/a	n/a	Physical examination	n/a	n/a	n/a	n/a	n/a	9.39	0.30
35	Normal	Male	52	n/a	n/a	Physical examination	n/a	n/a	n/a	n/a	n/a	32.72	1.98
36	Normal	Male	41	n/a	n/a	Physical examinatio	n/a	n/a	n/a	n/a	n/a	119.18	4.34

						n							
37	Normal	Female	20	n/a	n/a	Physical examination	n/a	n/a	n/a	n/a	n/a	71.03	0.47
38	Normal	Female	52	n/a	n/a	Physical examination	n/a	n/a	n/a	n/a	n/a	23.34	0.41
39	Normal	Male	37	n/a	n/a	Physical examination	n/a	n/a	n/a	n/a	n/a	28.11	0.50
40	Normal	Female	23	n/a	n/a	Physical examination	n/a	n/a	n/a	n/a	n/a	1.88	0.54
41	Normal	Female	55	n/a	n/a	Physical examination	n/a	n/a	n/a	n/a	n/a	15.52	1.07
42	Normal	Female	26	n/a	n/a	Physical examination	n/a	n/a	n/a	n/a	n/a	24.70	0.32
43	Normal	Male	25	n/a	n/a	Physical examination	n/a	n/a	n/a	n/a	n/a	21.09	2.81
44	Normal	Male	60	n/a	n/a	Physical examination	n/a	n/a	n/a	n/a	n/a	33.18	2.52
45	Normal	Female	27	n/a	n/a	Physical examination	n/a	n/a	n/a	n/a	n/a	16.19	0.60
46	Normal	Male	21	n/a	n/a	Physical examination	n/a	n/a	n/a	n/a	n/a	36.44	0.78
47	Normal	Female	24	n/a	n/a	Physical examination	n/a	n/a	n/a	n/a	n/a	15.08	0.24
48	Normal	Male	22	n/a	n/a	Physical examination	n/a	n/a	n/a	n/a	n/a	19.30	0.69
49	Normal	Male	28	n/a	n/a	Physical examinatio	n/a	n/a	n/a	n/a	n/a	24.93	0.30

						n							
50	Normal	Female	47	n/a	n/a	Physical examination	n/a	n/a	n/a	n/a	n/a	49.02	0.47
51	Normal	Male	20	n/a	n/a	Physical examination	n/a	n/a	n/a	n/a	n/a	31.10	2.33
52	Normal	Female	51	n/a	n/a	Physical examination	n/a	n/a	n/a	n/a	n/a	35.97	0.76
53	Normal	Female	50	n/a	n/a	Physical examination	n/a	n/a	n/a	n/a	n/a	71.53	0.91
54	Normal	Female	65	n/a	n/a	Physical examination	n/a	n/a	n/a	n/a	n/a	68.75	0.24
55	Normal	Female	20	n/a	n/a	Physical examination	n/a	n/a	n/a	n/a	n/a	19.08	1.75
56	Normal	Female	70	n/a	n/a	Physical examination	n/a	n/a	n/a	n/a	n/a	129.58	0.58
57	Normal	Male	66	n/a	n/a	Physical examination	n/a	n/a	n/a	n/a	n/a	82.05	n/a
58	Normal	Male	26	n/a	n/a	Physical examination	n/a	n/a	n/a	n/a	n/a	46.14	2.81
59	Normal	Female	19	n/a	n/a	Physical examination	n/a	n/a	n/a	n/a	n/a	18.85	0.17
60	Normal	Male	19	n/a	n/a	Physical examination	n/a	n/a	n/a	n/a	n/a	16.85	1.16
61	Normal	Female	38	n/a	n/a	Physical examination	n/a	n/a	n/a	n/a	n/a	61.00	3.46
62	Normal	Female		n/a	n/a	Physical examinatio	n/a	n/a	n/a	n/a	n/a	46.38	0.15

						n							
63	Normal	Female	20	n/a	n/a	Physical examination	n/a	n/a	n/a	n/a	n/a	27.66	0.39
64	Normal	Female	22	n/a	n/a	Physical examination	n/a	n/a	n/a	n/a	n/a	71.28	0.41
65	Normal	Male	42	n/a	n/a	Physical examination	n/a	n/a	n/a	n/a	n/a	42.32	0.41
66	Normal	Female	39	n/a	n/a	Physical examination	n/a	n/a	n/a	n/a	n/a	34.80	1.25
67	Normal	Male	25	n/a	n/a	Physical examination	n/a	n/a	n/a	n/a	n/a	66.49	0.65
68	Normal	Male	66	n/a	n/a	Physical examination	n/a	n/a	n/a	n/a	n/a	33.88	0.85
